# Spin Relaxation Does Not Preclude Magnetic Field Effects on Lipid Autoxidation

**DOI:** 10.1021/acscentsci.5c01229

**Published:** 2025-12-21

**Authors:** Gesa Grüning, Luca Gerhards, Chris Sampson, Daniel R. Kattnig, Ilia A. Solov’yov

**Affiliations:** † Institute of Physics, Carl von Ossietzky University, Carl-von-Ossietzky-Str. 9-11, 26129 Oldenburg, Germany; ‡ School of Physics, University of New South Wales, Sydney, New South Wales 2052, Australia; ¶ School of Biotechnology and Biomolecular Sciences, University of New South Wales, Sydney, New South Wales 2052, Australia; § Living Systems Institute, 3286 University of Exeter, Stocker Road, Exeter EX4 4QD, United Kingdom; ∥ Department of Physics, 3286 University of Exeter, Stocker Road, Exeter EX4 4QL, United Kingdom; ⊥ Research Center for Neurosensory Science, 11233 Carl von Ossietzky Universität Oldenburg, 26111 Oldenburg, Germany; # Center for Nanoscale Dynamics (CENAD), Institut für Physik, Carl von Ossietzky Universität Oldenburg, Ammerländer Heerstr. 114-118, 26129 Oldenburg, Germany

## Abstract

Spin correlations between radicals underpin key biological processes, and spin relaxation describes their decay due to environmental interactions. Radical pairs involving lipid peroxide radicals in bilayers have been proposed as a source of magnetic field effects (MFEs) in lipid autoxidation, but their viability has been questioned due to rapid relaxation in dynamic membranes. This study investigates whether MFEs can persist in lipid bilayers despite spin relaxation. Using an integrative approach combining all-atom molecular dynamics simulations, density functional theory (DFT) calculations, and spin dynamics modeling using Bloch–Redfield–Wangsness relaxation theory, we investigate a palmitoyl-linoleoyl-phosphatidylcholine (PLPC) model membrane containing 13ze-lipid peroxide radicals. We identify the peroxide group rotation and the lipid backbone dynamics as key drivers of spin relaxation. By computing g-tensors and hyperfine coupling constants via DFT and incorporating their molecular-dynamics-derived fluctuations into spin-dynamics simulations, we assess relaxation from hyperfine interactions, g-tensor fluctuations, and spin-rotational coupling. Our results demonstrate that MFEs persist in lipid bilayers despite thermal motion. Relaxation is dominated by g-fluctuations, which enhance MFEs at high magnetic fields. Surprisingly, our calculations also suggest possible MFEs in weak magnetic fields. These findings broaden the understanding of biological MFEs and highlight potential biomedical implications for ferroptosis, cancer, and oxidative stress-related diseases.

## Graphical abstract

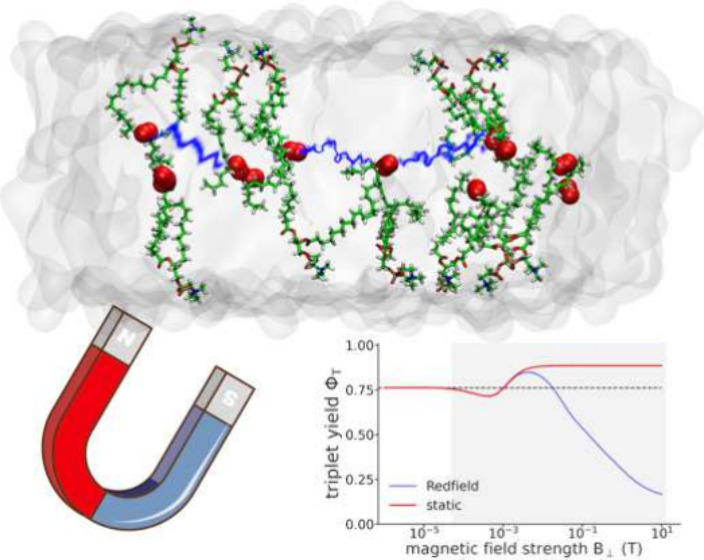

## Introduction

Lipid peroxidation is a well-studied process that has gained increasing attention due to its significance in biomedical applications.^1–15^ It involves the formation of lipid peroxide radicals through reactions with reactive oxygen species (ROS), such as hydroperoxyl radicals (HO_2_^•^), superoxide anions (O_2_^•–^), and hydroxyl radicals (OH^•^). These lipid peroxide radicals drive lipid autoxidation–a free radical chain reaction consisting of three stages: initiation, propagation, and termination.^1,2^ Termination occurs through pairwise recombination of radicals, a process that has been proposed to exhibit magnetosensitivity.^16^

Lipid peroxidation has been associated with several human diseases^104^ such as cancer,^7,10,17,18^ inflammation,^19,20^ diabetes,^21^ Alzheimer’s,^9,22^ and Parkinson’s disease^23,24^ and can also lead to ferroptosis, which is a form of regulated cell death.^25^

In this study, we explore the potential influence of magnetic fields on the recombination of lipid peroxide radicals. Specifically, we theoretically investigate the viability of magnetic field effects (MFEs) on the termination reactions of these lipids within a bilayer. Our analysis is based on the radical pair mechanism, which predicts magnetic field sensitivity in radical recombination due to spin dynamics. This mechanism has been widely applied to explain the influence of weak magnetic fields on various chemical reactions in biological systems.^26–28^ One of the most extensively studied examples is the biophysical basis of avian magnetoreception, attributed to radical pair dynamics in the photoreceptor protein cryptochrome.^29–37^

The radical pair (RP) formed by lipid peroxide radicals belongs to the class of F-pairs (freely diffusing radical pairs),^16^ in contrast to geminate radical pairs, which are relevant in magnetoreception. In an F-pair, the two radicals are generated independently,^38–40^ resulting in random relative spin orientations. Statistically, F-pairs thus generate the singlet and triplet electronic spin states with probabilities of 1/4 and 3/4, respectively (see Figure 1). This distribution reflects thermal equilibrium and, in itself, is not expected to be magnetosensitive in weak magnetic fields. However, upon encounter, the radicals may react if their total spin state allows. If singlet-state pairs recombine while triplet-state pairs remain nonreactive, a residual spin correlation can emerge. In these remaining spin-correlated radical pairs, the interconversion between singlet and triplet states is driven by hyperfine couplings (hfcs).^16^ This process becomes sensitive to an applied magnetic field through the Zeeman interaction, provided the spin correlation persists long enough, which is the question to be addressed here.

**Figure 1:**
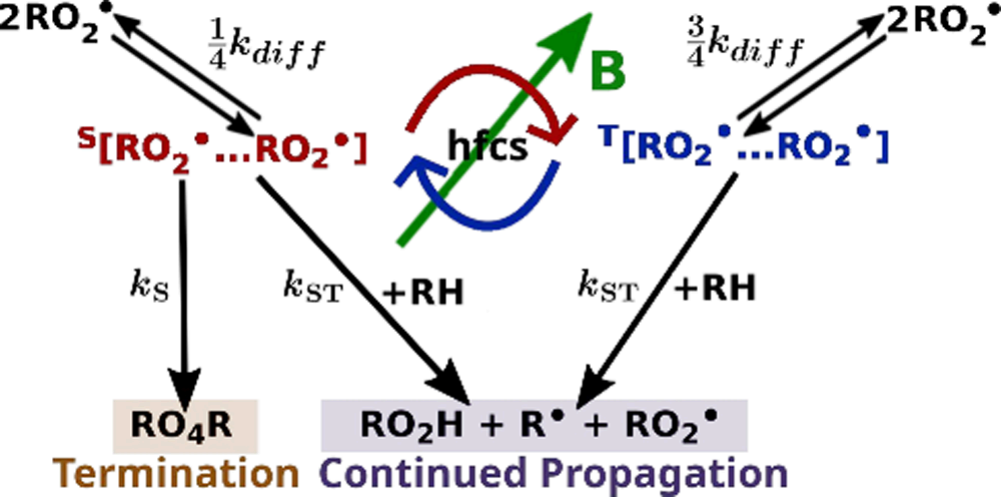
Reaction scheme for the radical pair recombination reaction, potentially leading to magnetic field effects on lipid peroxidation. **R** signifies the lipid without the peroxide group and the reactants in the square brackets form the radical pair. The superscripts at the radical pairs indicate the singlet (S) and triplet states (T) of the pair. The interconversion between the singlet and the triplet states is mediated by the hyperfine couplings (hfcs) and is sensitive to external magnetic fields. The F-pair is formed in diffusive encounters on the average yielding a singlet state in 1/4 and triplet states in 3/4 of the encounters, i.e., with rate constant $\frac{1}{4}k_{diff}$ and $\frac{3}{4}k_{diff}$ ,^16^ respectively. The singlet state of the RP can undergo an exclusive recombination reaction with the rate constant *k*_S_ = 2 × 10^8^ s^–1^ that leads to the termination of the lipid autoxidation process.^16^ Alternatively, continued propagation of lipid peroxidation can proceed from both the singlet and the triplet states of the RP with the rate constant *k*_ST_.

Figure 1 illustrates a possible radical pair reaction scheme involving two lipid peroxide radicals.^16^ The RP is assumed to form when two lipid peroxide radicals diffuse into close proximity, partially recombining to create a spin-correlated pair. From this RP state, two distinct reaction pathways are possible.^16^ In one pathway, the lipid peroxide radicals form a bond at their peroxide groups. Since bond formation requires the two electron spins to be antiparallel, this reaction can proceed only if the RP is in the singlet state. The resulting product, the singlet reaction yield, is diamagnetic, i.e., nonradical, effectively terminating the lipid peroxidation chain reaction. The alternative reaction pathways are spin-independent and allow the lipid peroxidation to continue. By contrast, the triplet reaction yield refers to the reaction product formed from radical pairs occupying one of the three triplet spin states. In Figure 1 this is represented by the right-hand reaction branch, where the triplet channel results in continued propagation of lipid peroxide radicals. During the continued propagation phase of lipid peroxidation, the total amount of lipid peroxide radicals is either preserved or increased, with an increase occurring if the conditions for chain ignition or degenerate chain branching are met.^41^ The number of lipid peroxide radicals that become subject to the continued propagation phase may thus be influenced by the external magnetic field, which alters the population in singlet and triplet states at the stage of the RP.^16^ Even small changes in the singlet–triplet ratio could lead to significant changes in radical concentration–potentially up to 3 orders of magnitude.^16,42^ In the present study, magnetic field effects (MFEs) are defined as a change of the triplet reaction yield induced by the presence or change of strength of an external magnetic field.

A measurable change in radical concentration in response to an external magnetic field serves as an experimental confirmation of the MFEs.^43^ However, the experimental study is difficult, prone to confounders, and the number of high-quality studies is still small. Several studies have focused on oscillating magnetic fields, in particular in the extremely low frequency (ELF) region. For short RP lifetimes, such low frequency external magnetic fields resemble the action of static fields.^27^ ELF fields were found to influence the oxidative status for fields above 1 mT^44^ and ELF were observed to affect the amount of ROS in the biological system.^45,46^ Whether the amount of ROS is increased or decreased varies with the studied biological system and the external field strength^47^ and frequency.^45^ Experimentally, magnetic fields have been observed to induce both cell apoptosis or proliferation depending on the specifics of the experiment such as cell type, type of magnetic field (static, low-frequency fields or a combination of a static and a low-frequency field), and the specific frequency of the field, and the pretreatment of cells (such as apoptosis-inducing drugs, heating or X-ray irradiation).^46,48–55^

This study investigates whether MFEs can persist in a lipid bilayer when spin relaxation due to lipid thermal motion is considered. Specifically, we analyze key dynamical parameters that affect spin relaxation in the radical pair (RP). The primary factors identified were the dihedral angle Ω, which quantifies the rotation of the peroxide group (see Figure 2B), and the motion of the radicals relative to the external magnetic field. Additional parameters related to peroxide group diffusion and spin-rotational relaxation were examined but found to have a negligible impact on RP reaction yields.

**Figure 2:**
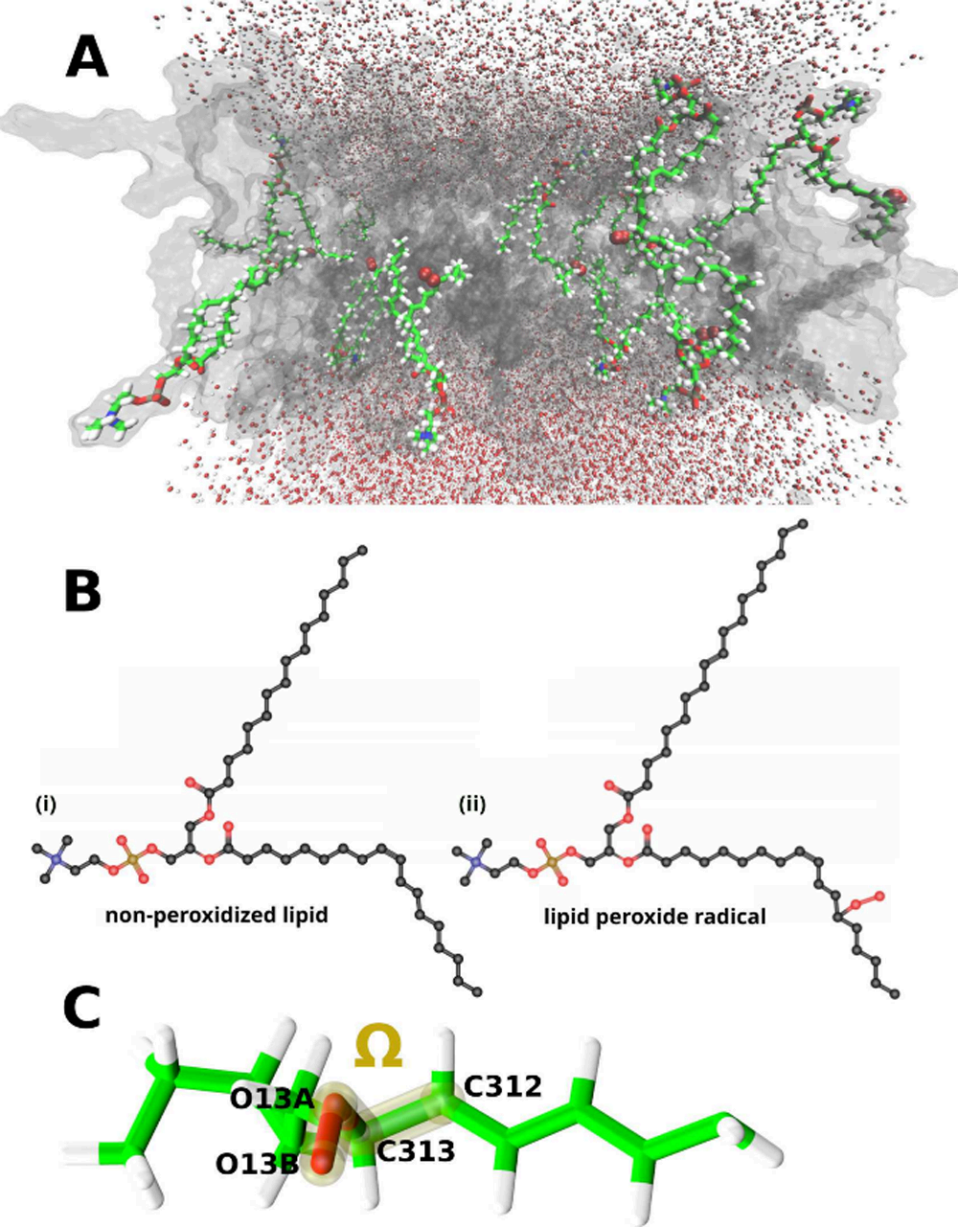
(A) Schematic representation of lipid peroxide radicals in a lipid bilayer (transparent gray surface) surrounded by a water box. The simulation included 255 lipids, whereby 12 of the lipids are lipid peroxide radicals with their peroxide group shown as red spheres. (B) The chemical structure of the nonperoxidized lipid (i) and the lipid peroxide radical (ii). Carbon nuclei are shown in black, nitrogen in blue, oxygen in red, and phosphorus in ochre. Hydrogen nuclei are not explicitly shown. The structure name for the nonperoxidized lipid is 1-palmitoyl-2-linoleoyl-*sn*-glycero-3-phosphocholine (PLPC). In the lipid peroxide radical (ROO^•^), a peroxide group OO^•^ is attached to the C313 nucleus of 1,2-dioleoyl-*sn*-glycero-3-phosphocholine (DOPC). The stereochemistry of the hydrogen nuclei surrounding the peroxide group defines the lipid peroxide radical as the 13ze isomer. (C) Only the selection of atoms used for the DFT calculations of the lipid peroxide radicals is shown. The rotation of the peroxide group (colored red) is characterized by the dihedral angle, Ω, spanned by the O16A, O16B, C313, and C312 atoms.

The motion of the lipid peroxide radicals influences both the hyperfine couplings of nearby nuclei and the g-tensors of the lipid peroxide radicals. The time-averaged hyperfine and Zeeman interactions modulate the yield of the magnetic field-dependent reaction in Figure 1 without directly inducing spin relaxation (henceforth referred to as the static case). However, fluctuations in hyperfine tensors and anisotropic g-tensors disrupt spin coherence, serving as potential pathways for spin relaxation. Here, we evaluated the efficiency of various spin relaxation mechanisms in lipid bilayers. Our findings indicate that RP systems in lipid peroxide radicals exhibit a dominant relaxation pathway driven by motion-induced modulation of the g-tensor. Crucially, we establish that significant MFEs persist in lipid bilayers even in weak magnetic fields, despite the presence of spin relaxation. Furthermore, we identify a range of external magnetic field strengths and conditions under which MFEs should be experimentally detectable.

## Methods

The study used a comprehensive approach, including all-atom molecular dynamics (MD) simulations, density functional theory (DFT) calculations, and spin dynamics calculations based on Bloch–Redfield-Wangsness (BRW) relaxation theory. First, the influence of lipid peroxidation on selected dynamic parameters was examined using MD simulations. The hyperfine interactions and g-tensors of the radicals were then calculated using DFT. Next, MFEs in the bilayer were evaluated by determining the chemical reaction yields using spin dynamics calculations for the static case. Finally, BRW relaxation theory was used to investigate how the motions observed in the MD simulation lead to spin relaxation via different relaxation mechanisms and whether the MFEs persist once the spin relaxation effects are included in the spin dynamics calculations.

### Model System

The lipid bilayer model in this study was based on 1-palmitoyl-2-linoleoyl-*sn*-glycero-3-phosphocholine (PLPC), which belongs to the class of phosphatidylcholine (PC) lipids. PC lipids are the most abundant lipids in animal and human cell membranes.^56^ The system studied consisted of a lipid bilayer that included a total of 255 lipids in the two leaflets, of which 12 were radicalized to form lipid peroxide radicals. The chemical structures of the nonperoxidized PLPC and the lipid peroxide radical are shown in Figure 2B. The lipid peroxide radicals included a peroxide group attached to the linoleoyl tail at the C313 carbon atom (see Figure 2B and C). The stereochemistry of the double bonds adjacent to the peroxide group was chosen as z and e, making the lipid peroxide radical the 13ze isomer (see Figure 2B). Throughout this work, the term ”lipid peroxide radical” refers to the whole radicalized lipid with the structure shown in Figure 2B (ii) and ”peroxide group” refers to only the OO^•^ substituent at the C313 atom.

### MD Simulations

All-atom molecular dynamics (MD) simulations employed the CHARMM force field,^57^ with parametrizations specific to lipids from the literature^58,59^ and, for the radical, created with the Force Field ToolKit^60^ and the Molefacture plugin for VMD.^61,62^ For the investigation of the dynamical parameters in the current study, the last 1,360 ns of a 5,000 ns trajectory were chosen to ensure that the system was well equilibrated. The NAMD calculations^63,64^ employed a Langevin thermostat^65^ to control the temperature to *T* = 310 K. The system consisted of 93,210 atoms, including 255 PC lipids in a bilayer and the surrounding water box. The equilibrated size of the simulation box was 91.4 Å × 91.4 Å × 108.1 Å. In the course of the simulation, the coordinates of all atoms in the lipid peroxide radicals were written out every 1 ps. A small write-out time interval was necessary to capture the fast components of structural dynamics and obtain more reliable fits of the relevant time correlation functions.

The dynamic properties of different parameters were observed using VMD.^62^ The most relevant parameter for the subsequent calculation of spin relaxation was the rotation of the peroxide group quantified by the dihedral angle Ω (see Figure 2B). Other investigated parameters included the distance between the two tails of each lipid peroxide radical and the lateral diffusion of the radicals.

### DFT-based calculations

#### MD-Averaged Hyperfine Coupling Tensors and g-Tensors

To derive the hyperfine interactions and the g-tensors for the peroxide groups in each lipid peroxide radical, selections of 32 atoms including the peroxide group and atoms in its vicinity were excised from the MD trajectory (see Figure 2C). The carbon–carbon bond that was cut in this process was capped with a hydrogen atom added using VMD. A total of 1008 geometries were extracted using VMD (84 structures for each of the 12 lipid peroxide radicals spaced equally over the simulation time of 1,360 ns) and used as input for quantum chemistry calculations with Gaussian16.^66^ The DFT-based calculations were undertaken as a two-step procedure: First, a geometry optimization was performed at the B3LYP/EPR-III level for the newly added H atoms.^67^ All other atoms were fixed during this step. In the second step, the hyperfine coupling tensors and g-tensors were calculated at the B3LYP/EPR-III level. EPR-III basis functions are purpose-built for the calculation of hyperfine tensors^67^ and were also used for the optimization of structures to ensure consistency. The total charge of the fragment was set to zero and the multiplicity was set to 2. The average hyperfine and g-tensors were calculated from the 1008 representative structures and were then used for the static spin dynamics calculations.

#### Time Evolution of the Hyperfine and g-Tensors

To calculate the spin relaxation effects, the time correlation functions of the pertinent spin Hamiltonian parameters, including auto- and cross-correlations of individual parameters, such as elements of the hyperfine tensors and g-tensor, are required. It is computationally infeasible, or at least impractical, to explicitly perform a DFT-based calculation of hyperfine and g-tensors for all 16.32× 10^6^ structures under consideration (12 lipid peroxide radicals at 1,360,000 time instances delivered from the MD simulations). Therefore, a mapping procedure developed in previous studies was employed.^34,68^ Following this protocol, quantum chemistry calculations are performed for structures, where selected geometric parameters are scanned. In the present study, the most crucial parameter was identified as the dihedral angle Ω (see Figure 2B), which was varied in steps of 1 degree within the range of values assumed during the MD simulations. The values of Ω and fragment orientation, as occurring in the 1,360,000 time instances in the MD simulation for each lipid peroxide radical, were extracted, and the relevant hyperfine and g-tensors were determined by matching with the DFT-calculated parameters while considering both the dihedral angle and fragment orientation. This procedure delivers a trajectory of hyperfine tensors and g-tensors in the reference frame of the MD simulation for each lipid peroxide radical. The variability and time correlation of fluctuations in the hyperfine tensors and g-matrices, which is central to spin relaxation, were determined through autocorrelation and cross-correlation functions of the hyperfine and g-tensor components.

### Spin Dynamics Calculations

Spin-dynamics calculations were performed with the Molspin software package,^69^ using its recent update that allows spin relaxation.^70,71^ In the following, static refers to results computed using the averaged spin Hamiltonian parameters without relaxation effects. These static calculations are compared with the results obtained when relaxation effects are considered in the BRW framework. In the present study, the spin selective recombination reaction from the singlet state (see Figure 1) was assumed to occur with a rate constant *k*_S_ of 2 × 10^8^ s^–1^ in line with earlier investigations.^16^ The spin-independent reaction leading to continued propagation was assumed to proceed with a rate constant of *k*_ST_ from the singlet or triplet state of the RP. *k*_ST_ was varied from 10^5^ s^–1^ to 10^9^ s^–1^ to sample different possible coherence lifetimes of the RPs.

The Hilbert space of the spin system is constructed as the tensor product of the two electron spin-1/2 spaces (one for each lipid peroxide radical in the RP) with the nuclear spin spaces of all coupled nuclei:

$$H=H_{e_{1}}⊗H_{e_{2}}⊗({⊗}_{i=1}^{2}{⊗}_{n_{i}=1}^{N_{i}}H_{I_{i,n_{i}}})$$ 1

The electronic subspace $H_{e_{1}}⊗H_{e_{2}}$ is 4-dimensional and is spanned by the singlet {|*S*⟩} and triplet {|*T*_+_⟩, |*T*_0_⟩, |*T*_–_⟩} states. Each radical i is further coupled to *N*_*i*_ nuclear spins (indexed by *n*_*i*_ = 1,...,*N*_*i*_), each of dimension (2*I*_*i*,*n*_*i*__ + 1), so that the total Hilbert space has the dimension

$$\dim\left(H\right)=4\times \prod_{i=1}^{2}\prod_{n_{i=1}}^{N_{i}}\left(2I_{i,n_{i}}+1\right)$$ 2

The density operator *ρ̂*(*t*) acts on the full space H . The triplet projection operator *P̂*_*T*_ is defined as

$${\mathrm{P̂}}_{T}=\left(\left|T_{+}\right\rangle \langle T_{+}|+\left|T_{0}\right\rangle \langle T_{0}|+\left|T_{-}\right\rangle \langle T_{-}|\right)⊗{Î}_{\mathrm{nuc}}$$ 3

where *Î*_nuc_ is the identity operator on the nuclear spin subspace. Thus, *P̂*_*T*_ acts only on the two-electron spin states, while leaving the nuclear spins unchanged. Taking the trace of *P̂*_*T*_ *ρ̂*(*t*) therefore gives the total probability the radical pair occupying any of the triplet states, irrespective of the nuclear configuration. The triplet quantum yield of the RP reaction was calculated as follows:

$$\Phi_{T}=k_{\mathrm{ST}}\int_{0}^{\infty }\mathrm{Tr}\left({\mathrm{P̂}}_{T}\mathrm{\rho ̂}\left(B,t\right)\right)\;dt$$ 4

Here, *k*_ST_ is the reaction rate constant from the triplet state, Tr denotes the trace, P̂_T_ is the projection operator onto the triplet states, and *ρ̂*(**B**, *t*) is the time-dependent density operator, which satisfies the stochastic Liouville equation,

−dρ̂(t)dt=iℏ[Ĥ,ρ̂(t)]+K^^ρ(t)+R^^ρ̂(t) 5

where *ℏ* is the reduced Planck’s constant and the Hamiltonian Ĥ is describing the static interactions driving the time evolution of *ρ̂*(**B**, *t*). The reaction superoperator K^^ accounts for the spin-selective reactivity and R^^ is the relaxation superoperator. In the present study, the Zeeman and hyperfine interactions were included in Ĥ, which leads to the following factorization:

$$Ĥ=\overset{2}{\underset{i=1}{\sum }}\left(\mu_{B}B\cdot g_{i}\cdot {Ŝ}_{i}+\underset{n_{i}}{\sum }{Î}_{i,n_{i}}\cdot A_{i,n_{i}}\cdot {Ŝ}_{i}\right)$$ 6

The index *i* iterates over the two radicals and **Ŝ**_*i*_ is the electron spin operator for radical *i*. The first term describes the Zeeman interaction of the radicals with the external magnetic field **B**, μ_B_ is the Bohr magneton and **g**_*i*_ is the g-matrix. The second term in eq 6 is the hyperfine interaction that describes the interaction of the *n*_*i*_-th nuclear spin **Î**_*i*,*n*_*i*__ with the electron spin in radical *i* through the hyperfine tensor **A**_*i*,*n*_*i*__. Assuming a negligible cross-relaxation, the relaxation superoperator R^^ in eq 5 can be expressed in terms of the contributions of different spin relaxation channels,

R^^=R^^hf+R^^g+R^^sr 7

where R^^hf accounts for the spin relaxation effects due to fluctuations in the hyperfine interaction tensors, R^^g is the contribution due to fluctuations in the g-matrix, and R^^sr accounts for spin relaxation due to the spin-rotational interaction. The different contributions to spin relaxation will be described in the following. In the interaction picture, R^^hf and R^^g can be expressed in terms of spin system operators ${Â}_{\alpha }$ and ${Â}_{\beta }$ , and the coupling between the spin system and the environment, which is expressed through the covariance functions *g*_*αβ*_ of interaction parameters. Specifically,

R^^ρ̂(t)=∑αβ∫0∞dτ(gαβ(τ)[Âα(t),Âβ(t−τ)ρ̂(t)]−gαβ*(τ)[Âα(t),ρ̂(t)Âβ(t−τ)]) 8

where τ is the correlation time lag and the indices α and β enumerate the 9 components of the hyperfine tensor or g-matrix and the associated spin operators, i.e. α, β in {*xx*, *xy*, ···}. The validity of the BRW approach was tested and confirmed in SI section S12.

#### Hyperfine Relaxation

The correlation functions of the hyperfine coupling operators were obtained as follows: the 3 × 3 hyperfine tensors **A**_*i*,*n*_*i*__, see eq 6, obtained via the aforementioned mapping procedure at 1,360,000 instances of time were used and the difference between the momentary hyperfine coupling **A**_*i*,*n*_*i*__(*t*) and the average hyperfine coupling **A̅**_*i*,*n*_*i*__ was calculated for each tensor component α at each time instance. It turned out that just one nucleus (*n*_*i*_ = 1) contributed significantly to the total hyperfine coupling in each radical *i*. Therefore, for brevity, the indices *n*_*i*_ and *i* used in eq 6 are omitted in the following. The auto- or cross-correlation functions of the difference to the average hyperfine coupling Δ*A*_α_ = *A*_α_(*t*) – *A̅*_α_ were calculated for each tensor component using the fast Fourier transform method. To obtain the correlation times of the hyperfine couplings to quantify the spin relaxation in the quantum yield calculations, the covariance functions *g*_*αβ*_^*h*^(τ) with the *h* superscript denoting the hyperfine interaction were fitted with a sum of 50 exponential decays of the form:

$$g_{\alpha \beta }^{h}\left(\tau \right)\approx cov_{\;\alpha \beta }^{h}\overset{50}{\underset{j=1}{\sum }}c_{\alpha \beta ,j}^{h}\;\exp\left(-\frac{\tau }{\tau_{j}}\right)$$ 9

The τ_*j*_ were spaced logarithmically between 0.5 ps and 100 ns. The *h* superscript is indicating variables that pertain to the hyperfine coupling and α and β ∈{*xx*, *xy*, *xz*, ···} denote tensor components in all of the following text. cov­(*A*_α_, *A*_β_) = cov_*αβ*_^*h*^ is the covariance of the hyperfine tensor components *A*_α_ and *A*_β_, τ is the lag time, and the *c*_*αβ*,*j*_^*h*^ were considered fitting parameters.

The relaxation superoperator in eq 8 can also be written in terms of the spectral densities *J*_*αβ*_^*h*^(ω) evaluated at the transition frequencies of the system ω.^34,72,73^ For every magnetic nucleus in the spin system, *J*_*αβ*_^*h*^(ω) can be expressed in terms of the hyperfine correlation functions *g*_*αβ*_^*h*^ defined in eq 9 as the correlation between the hyperfine tensor components *A*_α_ and *A*_β_:^34,70,73^

$$J_{\alpha \beta }^{h}\left(\omega \right)=\int_{0}^{\infty }g_{\alpha \beta }^{h}\left(\tau \right)\;\exp\left(i\omega \tau \right)\;d\tau$$ 10

For ω = 0, the spectral densities *J*_*αβ*_^*h*^(0) can thus be approximated as

$$J_{\alpha \beta }^{h}\left(0\right)\approx cov_{\alpha \beta }^{h}\overset{50}{\underset{j=1}{\sum }}c_{\alpha \beta ,j}^{h}\tau_{j}$$ 11

The covariances cov_*αβ*_^*h*^ are here used in units of (rad^2^/ns^2^) and the spectral densities *J*_*αβ*_^*h*^ in units of (rad^2^/ns). Finally, the effective correlation time of the hyperfine coupling τ^*h*^ of the hyperfine tensor components *A*_α_ and *A*_β_ can be approximated as

$$\tau^{h}\approx \overset{50}{\underset{j=1}{\sum }}c_{\alpha \beta ,j}^{h}\tau_{j}$$ 12

with the index *j* iterating through the 50 exponential contributions to the multiexponential fit of the covariance function *g*_*αβ*_^*h*^(τ), where each monoexponential correlation time τ_*j*_ is weighted by fitting factor *c*_*αβ*,*j*_^*h*^ subject to ∑ _*j* = 1_^50^ *c*_*αβ*,*j*_^*h*^ = 1.

#### g-Tensor Anisotropy Relaxation

The Zeeman term Ĥ_Z_ of the Hamiltonian in eq 6 can be written as

$${Ĥ}_{Z}=\overset{2}{\underset{i=1}{\sum }}\mu_{B}B\cdot g_{i}\cdot {Ŝ}_{i}=\overset{2}{\underset{i=1}{\sum }}\mu_{B}\left[\begin{array}{lll} B_{x} & B_{y} & B_{z} \end{array}\right]{\left[\begin{array}{lll} g_{xx} & g_{xy} & g_{xz}\\ g_{yx} & g_{yy} & g_{yz}\\ g_{zx} & g_{zy} & g_{zz} \end{array}\right]}_{i}{\left[\begin{array}{l} {Ŝ}_{x}\\ {Ŝ}_{y}\\ {Ŝ}_{z} \end{array}\right]}_{i}$$ 13

where μ_B_ is the Bohr magneton, **B** is the external magnetic field, **g**_*i*_ is the g-matrix and **Ŝ**_*i*_ is the spin vector with the index *i* enumerating the two radicals in the RP. For a magnetic field perpendicular to the lipid bilayer, $B=\left[\begin{array}{lll} 0 & 0 & B_{z} \end{array}\right]$ , eq 13 simplifies to

$${Ĥ}_{Z}=\overset{2}{\underset{i=1}{\sum }}\mu_{B}B_{z}{\left(g_{zx}{Ŝ}_{x}+g_{zy}{Ŝ}_{y}+g_{zz}{Ŝ}_{z}\right)}_{i}$$ 14

To calculate the relaxation caused by the g-tensor anisotropy, analogous to the procedure for the hyperfine coupling, the difference between the momentary g-tensor *g*(*t*) and the average g-tensor *g̅* was calculated for each tensor component *g*_α_ (with α = {*xx*, *xy*, ···}) at each time instance. The g-tensors were obtained in the reference frame of the MD simulations and *g̅* was averaged over the 12 lipid peroxide radicals. Autocorrelations and cross-correlations of the difference from the average g matrix Δ*g*_α_(*t*) = *g*_α_(*t*) – *g̅*_α_ were calculated for each component of the g-tensor. Analogous to the hyperfine spin relaxation, a multiexponential fit was applied to the correlation functions *g*_*αβ*_^*g*^(τ) of the g-tensor as

$$g_{\alpha \beta }^{g}\left(\tau \right)\approx cov_{\alpha \beta }^{g}{\left(\frac{\mu_{B}B_{z}}{\hbar }\right)}^{2}\overset{50}{\underset{j=1}{\sum }}c_{\alpha \beta ,j}^{g}\;\exp\left(-\frac{\tau }{\tau_{j}}\right)$$ 15

Here and in the following, the *g* superscript denotes variables pertaining to the g-tensor anisotropy. *cov*_*αβ*_^*g*^ = *cov*(*g*_α_, *g*_β_) is the covariance of the components α and β of the g-matrix, and the field was assumed to be aligned with the *z*-direction. Thus, α and β both involve the z-component label of the magnetic field. The τ_*j*_ values in eq 15 were logarithmically spaced between 0.5 ps and 100 ns; τ stands for the lag time, and the *c*_*αβ*,*j*_^*g*^ were the fitting parameters of the g-matrix correlation fits. The effective correlation time τ^*g*^ of the g-tensor components *g*_α_ and *g*_β_ can be approximated as

$$\tau^{g}\approx \overset{50}{\underset{j=1}{\sum }}c_{\alpha \beta ,j}^{g}\tau_{j}$$ 16

The spectral densities for the g-tensor relaxation at ω = 0 can be expressed as

$$J_{\alpha \beta }^{g}\left(0\right)\approx {\left(\frac{\mu_{B}B_{z}}{\hbar }\right)}^{2}\overset{50}{\underset{j=1}{\sum }}cov_{\alpha \beta }^{g}c_{\alpha \beta ,j}^{g}\tau_{j}$$ 17

#### Spin Rotational Relaxation

Relaxation due to spin rotational relaxation was estimated on the basis of a simple model. Assuming isotropic rotational diffusion, the associated relaxation rate *k*_*sr*_ is given by

$$k_{sr}\approx \frac{1}{9}\frac{\Delta g^{2}}{\tau^{\Omega }}$$ 18

where τ^Ω^ is the rotational correlation time associated with the spin-bearing component, i.e. the peroxide group, and

$$\Delta g^{2}=\Delta g_{xx}^{2}+\Delta g_{yy}^{2}+\Delta g_{zz}^{2}$$ 19

with Δ*g*_*nn*_ denoting the difference of the *n*th eigenvalue of the g-matrix from the free-electron g-factor *g*_*e*_ = 2.0013. Here, we estimate τ^Ω^ as the correlation time associated with the change in the cosine of the dihedral angle that describes the rotation of the peroxide group. This rotation is the fastest rotation in the considered system, suggesting that eq 18 provides an upper bound of the spin rotational relaxation rate. Finally, the effect of spin rotational relaxation is here included in the master equation using^70,74,75^

R^^srρ̂(t)=−ksr(32ρ̂−∑i∑kŜikρ̂(t)Ŝik) 20

where **Ŝ**_*ik*_ is the *k*-component (*k* = *x*, *y*, *z*) of the electron spin operator for the radical *i*.

## Results

This section presents the results of our calculations and is structured as follows: first, different relevant motions in the lipid bilayer are discussed, followed by the analysis of hyperfine interactions in the lipid peroxide radicals. Finally, calculations of MFEs with and without spin relaxation effects are shown.

### Analysis of Motions in the Lipid Bilayer

#### Comparing the Dynamics of Peroxidized and Nonperoxidized Lipids

Comparisons in dynamics between lipids with and without the peroxide group allow assessing the influence of the peroxide group on lipid movement. The motion of the peroxide group relative to the orientation of the external magnetic field induces spin relaxation. Most previous experimental and analytical studies investigated the motion of nonperoxidized lipids.^76–78^ It is therefore interesting to investigate how the specific motions of nonperoxidized lipids differ from those of the 13ze-lipid peroxide radical.

One of the parameters in which the presence of a radical is likely to have an impact is the distance between the two lipid tails. This can be attributed to steric effects that arise in the lipid peroxide radical compared to the nonperoxidized lipid variant due to the addition of the peroxide group. Additionally, movement in the lipid tails changes the orientation of the radicals and modulates the spin Hamiltonian parameters, which leads to spin relaxation.

As illustrated in Figure 3, the two lipid-tail distances (at the tail end (*d*_316_) and at the height of the peroxide group (*d*_313_)) are defined, and their distributions are compared in Figure 4. The comparison was performed for lipid peroxide radicals and the nonperoxidized lipid.

**Figure 3:**
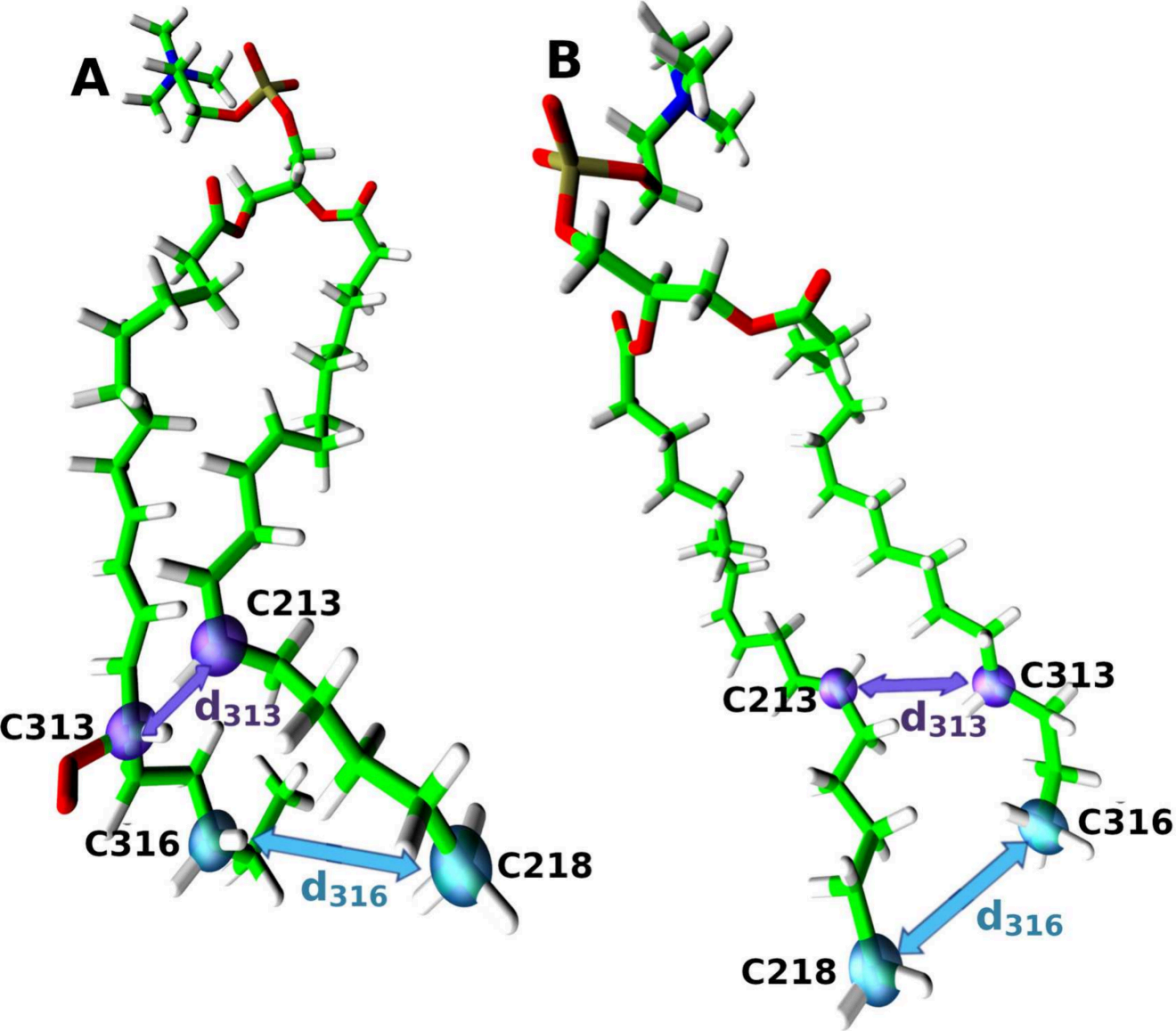
Characteristic distances between lipid tails. Panel (A) illustrates the measurements in lipid peroxide radicals, while (B) shows the distances measured in nonperoxidized lipids. Light blue arrows indicate the distance *d*_C316_, measured between the carbon nuclei C218 and C316, which are the carbon atoms furthest from the headgroup. The smaller purple arrows mark the distance *d*_C313_ at the height of the peroxide group, which is attached to the C313 carbon nucleus in the lipid peroxide radical.

**Figure 4:**
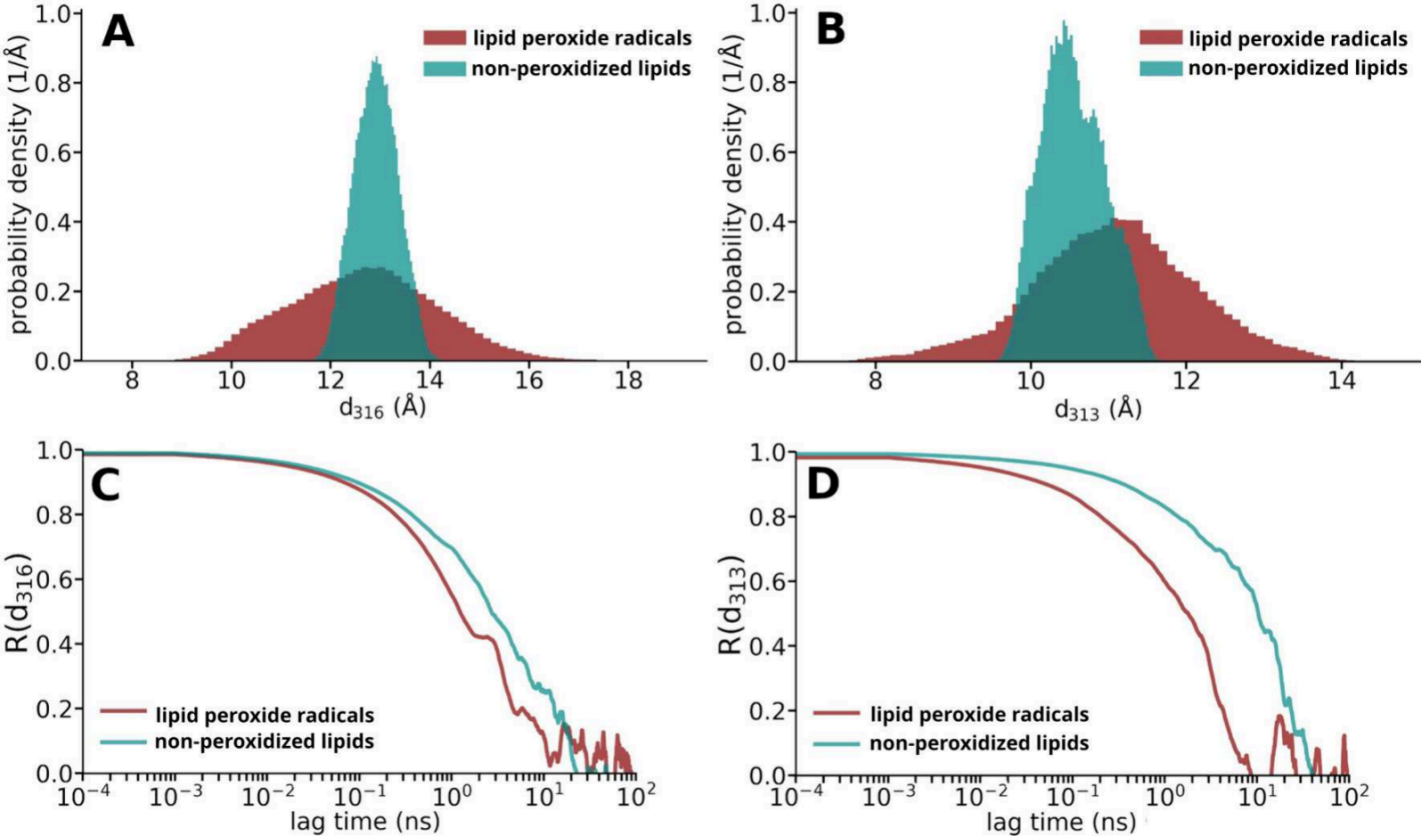
Distributions of the probability densities of the distances *d*_313_ and *d*_316_ (see Figure 3) between the two lipid tails in lipid peroxide radicals (dark red) and nonperoxidized lipids (petrol) (A and B). The variance of the distribution for lipids with a peroxide group is noticeably larger. Comparison of the autocorrelation functions *R* of the tail-to-tail distances *d*_316_ and *d*_313_ in lipids with and without a peroxide group (C and D).

The variance of the tail-to-tail distance is much larger for the lipid peroxide radicals (see Figure 4A and B). On average, the lipid tails are slightly closer together for the lipid peroxides at the height of the peroxide group (see Figure 4B).

Figure 4C and D shows a comparison of the mobility of the lipid tails with and without a peroxide group. For this purpose, the normalized autocovariance function *R*(τ) of the distance between the lipid tails is shown. The faster the autocovariance function drops to zero, the faster the motion loses correlation. The tail-ends (Figure 4C) of lipids with and without the peroxide group move similarly. At the height of the peroxide group (Figure 4D) the lipid peroxide radicals are much more mobile than the nonperoxidized lipids. Visually, such behavior may be observed in the MD simulations, where the tails of the peroxide lipids curl up more than the nonperoxidized lipids.

#### Rotation of the Peroxide Group

The dihedral angle Ω that describes the rotation of the peroxide group is formed by the atoms O16A, O16B, C313, and C312 (see Figure 2B). The distribution functions of the dihedral angles were calculated to assess whether the MD simulations provided sufficient statistical sampling, allowing the average distribution of Ω across all lipids to be considered representative of each individual peroxide lipid.

Simulations revealed that the peroxide group did not assume a position where the O13A–O13B bond appeared within the region spanned by the angle formed by the atoms C312, C313, and C314; this behavior was the same for the 12 peroxide lipids (see Figure 5A). The probability density distributions of the dihedral angle Ω, evaluated for the 12 different peroxide lipids in Figure 5A, appear very similar, further strengthening the rationale for using Ω as the key parameter in describing the spin dynamics of the lipid peroxide radicals.

**Figure 5:**
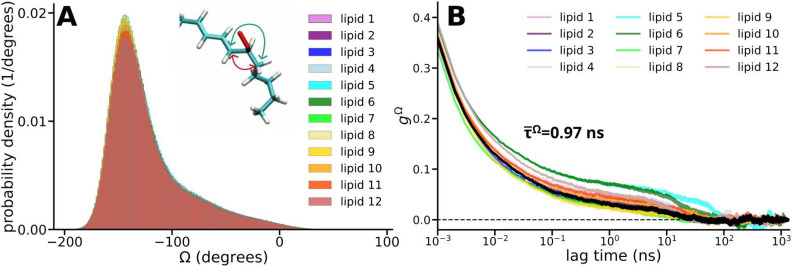
Probability density distributions of the dihedral angles Ω measured in the 12 peroxide lipids at 1,360,000 time instances every 1 ps for every lipid (A). The red arrow in the inset shows the parameter range that the dihedral angle does not sample. The normalized autocorrelation functions *g* ^Ω^ of cos­(Ω) for the respective lipids (B). The black line shows the average autocorrelation curve of the 12 lipid peroxide radicals from which the average correlation time of the rotation of the peroxide group *τ̅* ^Ω^ was obtained.

The fluctuations in the dihedral angle Ω were further assessed by the autocorrelation function *g* ^Ω^ of cos­(Ω) (see Figure 5B). The correlation time of Ω is an important parameter for all spin relaxation channels investigated in the present study. The average effective correlation time of the 12 peroxide lipids turns out to be 0.97 ns, while the overall shape of *g* ^Ω^ looks similar for all lipid peroxide radicals, and therefore, the average autocorrelation function is a good unified measure for the entire population.

### Hyperfine Couplings

#### MD-Averaged Hyperfine Couplings

Figure 6 shows the MD-averaged hyperfine interactions of the magnetic nuclei in the surroundings of the peroxide group. The strongest hyperfine interactions arise for the H13X nucleus adjacent to the peroxide group (atoms O13A and O13B). The average hyperfine coupling of H13X is close to isotropic due to the thermal motion of the lipid, which leads to averaging of the anisotropic hyperfine tensor contributions, which are evident for individual time instances (see Figure 6A).

**Figure 6:**
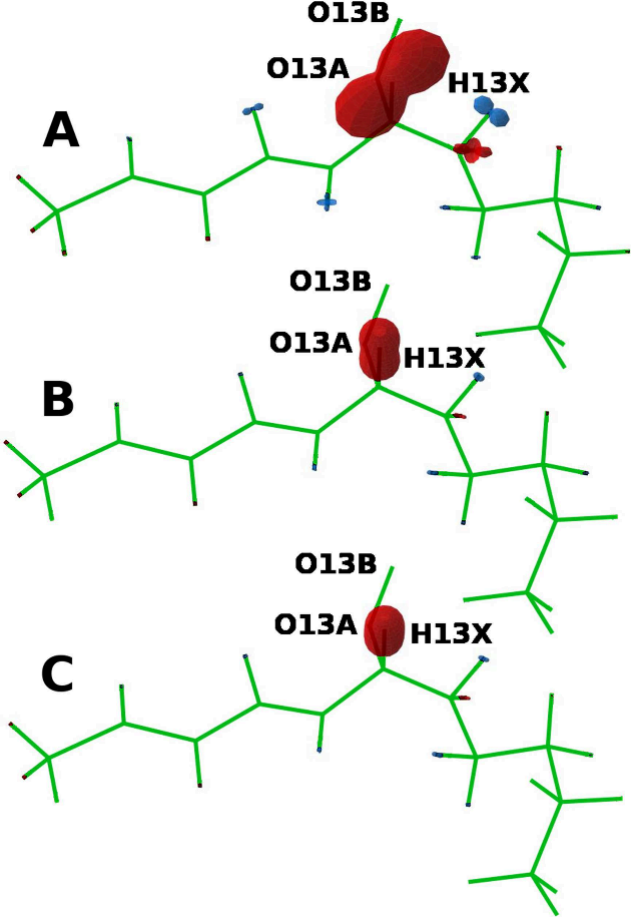
Hyperfine coupling tensors in one peroxide lipid at one time instance (A); only interactions of hydrogen nuclei are displayed. Red (blue) lobes indicate a positive (negative) value of the trace of the hyperfine coupling tensor. Average hyperfine coupling tensors computed for one lipid and averaged over 84 time instances spread over 1,360 ns (B). Hyperfine interactions averaged over all 12 peroxide lipids and the 84 time instances (C).

Figure 6C furthermore reveals that the hyperfine interactions of all nuclei other than H13X turn out to be insignificant, suggesting that the spin dynamics of the studied RP can be adequately modeled in terms of two isotropically coupled H13X nuclei, one in each radical. The average hyperfine coupling has been calculated as the average over the entire trajectory and the 12 peroxide lipids.

### Correlation Times of Interactions

#### Hyperfine Coupling Correlation Time

The angle Ω is the most important parameter contributing to hyperfine spin relaxation. To quantify the relaxation effect caused by the temporal changes in the hyperfine coupling tensor of H313X, the tensors were reconstructed for every time instance in the MD simulation using the mapping procedure described earlier. The auto- and cross-correlation functions of the different tensor components of the hyperfine coupling tensor were then calculated and analyzed.

Figure 7 shows an exemplary autocorrelation function of the A_*xx*_ component of the hyperfine coupling tensor of the H313X nucleus in the reference frame of the MD simulation. The autocorrelation functions of the individual peroxide lipids and the average autocorrelation function are shown. Similar plots are obtained for the remaining 20 distinct auto- and cross-correlation functions resulting from the other components of the 3 × 3 hyperfine tensors of the H313*X* nucleus. Only 21 of the correlation functions are distinct because permutations of hyperfine tensor components result in the same correlation functions, e.g. *g*_*xx*,*xy*_^*h*^ = *g*_*xx*,*yx*_^*h*^ = *g*_*xy*,*xx*_^*h*^ = *g*_*yx*,*xx*_^*h*^. The correlation functions of the hyperfine coupling tensor components were fitted with the sum of exponential functions defined in eq 9 and the resulting fits were averaged to reconstruct the averaged correlation times needed for the spin dynamics calculations. The average correlation time *τ̅* ^*h*^ for the *A*_*xx*_ autocorrelation function shown in Figure 7 turned out to be 0.92 ns. The effective auto- and cross-correlation times for all other hyperfine tensor components are given in Table S5 in the Supporting Information (SI).

**Figure 7:**
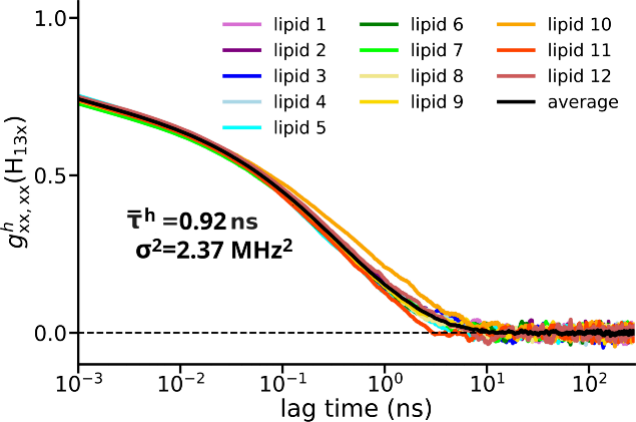
Normalized autocovariance function *g*_*xx*,*xx*_^*h*^ of the A_*xx*_ component of the hyperfine tensors computed separately for the 12 lipid peroxide radicals and the average autocorrelation function (black line). *τ̅* ^*h*^ depicts the value of the average effective correlation time obtained by fitting each of the individual autocovariance functions with eq 9, calculating the effective correlation times τ^*h*^ for the individual lipids using eq 12 and averaging the resulting effective correlation times. σ^2^ is the variance of the A_*xx*_ hyperfine tensor components measured over the entire MD simulation and then averaged over the 12 lipid peroxide radicals.

#### g-Tensor Anisotropy Correlation Time

Analogously to the procedure described previously for calculating the correlation functions of the hyperfine tensor component, the 21 distinct auto- and cross-correlation functions were calculated for the components of the 3 × 3 g-tensor. Figure 8 shows an exemplary autocorrelation function of the g_*xx*_ component of the g-tensors of the different lipid peroxide radicals. The correlation functions were fitted with a sum of exponential functions to obtain the fitting factors (*c*_*αβ*,*j*_^*g*^) needed to construct the spectral densities for the spin dynamics calculations; see eq 17. The average effective correlation time *τ̅* ^g^ = 1.21 ns obtained for the *g*_*xx*_ component of the g-tensor anisotropy turned out to be slightly longer than the effective correlation time for the hyperfine interaction *τ̅* ^*h*^ = 0.92 ns calculated for the A_*xx*_ hyperfine tensor component (see Figure 7). The effective auto- and cross-correlation times for all other g-tensor components are given in Table S6 in the Supporting Information.

**Figure 8:**
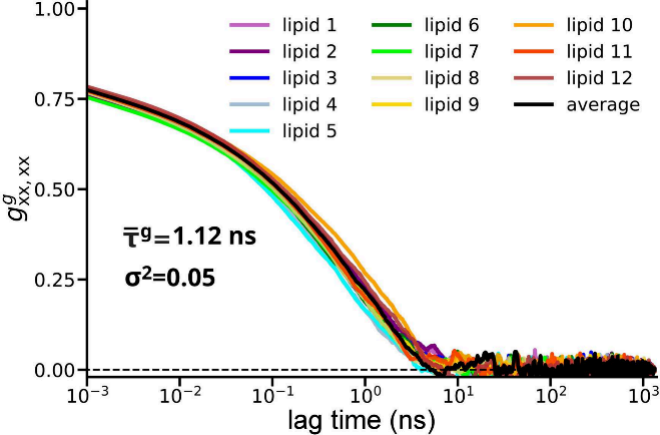
Autocorrelation function *g*_*xx*,*xx*_^*g*^ of the g_*xx*_ component of the g-tensors computed separately for the 12 peroxide lipids and the average autocorrelation function (black line). *τ̅* ^*g*^ provides the value of the average effective correlation time (see eq 16), obtained by fitting each of the individual autocovariance functions with eq 15 and averaging the resulting effective correlation times τ^*g*^. σ^2^ is the variance of the g_*xx*_ g-tensor components measured over the entire MD simulation time for the individual peroxide lipids and then averaged.

### Magnetic Field Effects

The postulated magnetic field-sensitive recombination of lipid peroxide radicals,^16^ hinges on the conservation of spin coherence for a time period sufficient for the applied field to create an impact, i.e., the coherent lifetime of a RP must be comparable to or exceed the Larmor precession frequency of the electron precession in the external magnetic field. In the present study, the effect of dynamic degrees of freedom on the spin evolution, and thus the propensity to form MFEs, was assessed employing the BRW theory.^70^ In the present investigation, we define MFEs as changes in the triplet reaction yield (see Figure 1) induced by the presence and strength of an external magnetic field. A reduced triplet yield relative to the zero-field case, or relative to another field strength, implies a lower steady-state concentration of lipid peroxide radicals in the bilayer. This outcome arises because a larger fraction of radical pairs undergo recombination via the spin-selective singlet channel, forming covalent bonds at the peroxide groups. Subsequent chemical steps, such as water abstraction and bond cleavage, then yield two nonradical lipid products, thereby depleting the radical population. Conversely, an increased triplet yield in the presence of a magnetic field constitutes an MFE leading to enhanced persistence of lipid peroxide radicals within the bilayer.

#### Influence of Spin Relaxation on the Triplet Yield

Figure 9 illustrates the magnetic field dependence of the triplet yield of a peroxide lipid RP assumed to be generated in the triplet state, as illustrated in Figure 1. Here, the magnetic field is applied perpendicular to the membrane surface. Figure 9 shows that lipid recombination is generally predicted to be magnetosensitive.

**Figure 9:**
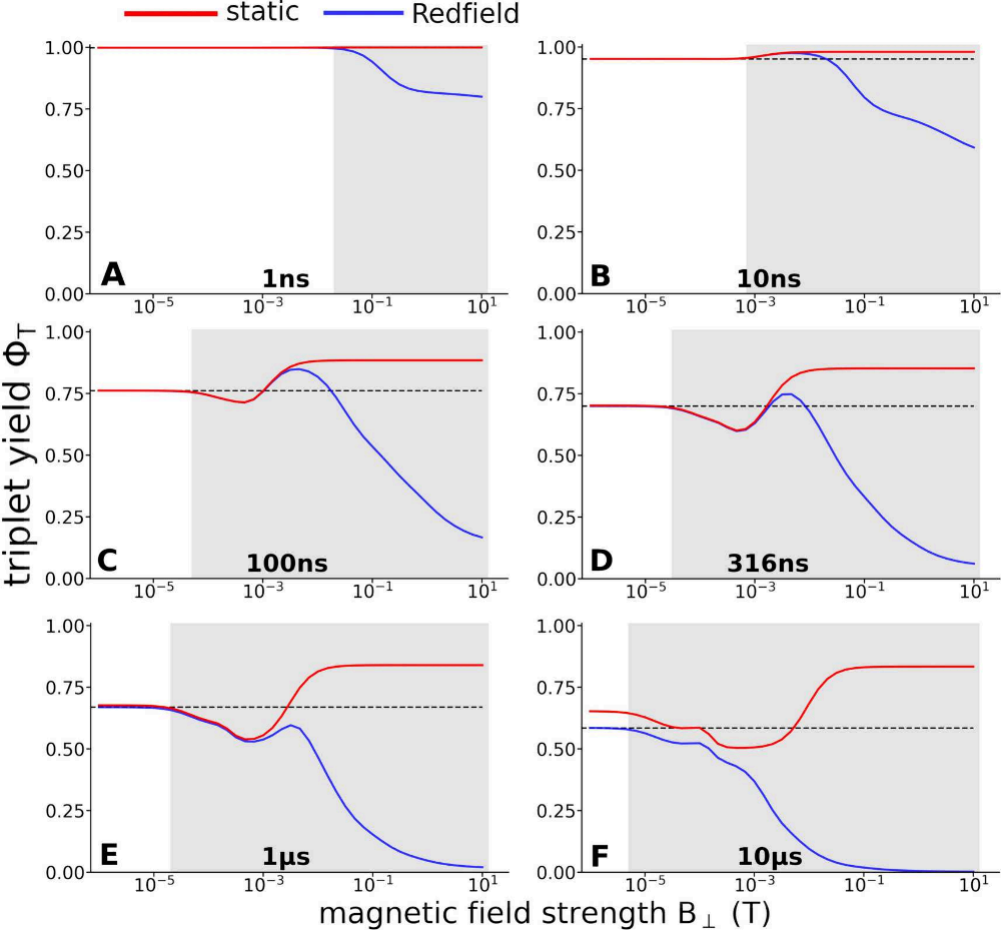
Predicted MFE for one RP arising in the lipid bilayer with hyperfine interactions modulated by the H313X nuclei without spin relaxation (static) and with spin relaxation accounted for via BRW-theory (Redfield). The magnetic field was applied perpendicular to the membrane surface. The subplots A–F show the results of triplet yield Φ_T_ calculations for a RP with six different lifetimes controlled through the rate constant *k*_ST_ (see Figure 1: 1 ns (A), 10 ns (B), 100 ns (C), 316 ns (D), 1 μs (E), 10 μs (F)). The black dashed line shows the triplet yield in the absence of a magnetic field. The gray area marks the field strengths for which MFEs could be observable in the system with spin relaxation.

In the absence of spin relaxation (static), the magnetic field dependence of the triplet yield exhibits the typical bimodal behavior due to the low-field effect at about 1 mT and leveling-off at high field, in line with the hyperfine mechanism.^79,80^ Spin relaxation reduces the triplet yield, whereby the effect is particularly pronounced in higher magnetic fields. Regardless of the lifetime of the RP, relaxation enables large MFEs in high fields as a result of the g-anisotropy relaxation mechanism at high magnetic fields (see Figure 9A–F). For intermediate lifetimes, the combination of the hyperfine mechanism and fast spin relaxation at high fields gives rise to a comparably complex magnetic field dependence of the recombination yield, with a trough followed by a peak around the magnetic field intensity of 1–10 mT (see Figure 9C and D). For RP lifetimes of around 1 μs, the peak at the intermediate field is suppressed, leaving the triplet yield at 10 mT below the yield value in the absence of a magnetic field (dashed line Figure 9E). For even longer lifetimes (10 μs), the magnetic field dependence of the recombination yield resembles that predicted in the absence of spin relaxation but shifted to lower yields up to a field strength of about 1 mT (see Figure 9F). For higher magnetic field strengths, spin relaxation completely suppresses the triplet yield, as the initial radical pair is quickly relaxed into a recombining singlet state.

In general, the findings demonstrate that MFEs are expected for all RP lifetimes (see gray-marked regions in Figure 9). Spin relaxation does not suppress the magnetic field sensitivity of an RP reaction in lipid peroxide radicals but instead greatly enhances the MFEs in high magnetic fields. Importantly, MFEs are expected in weak magnetic fields, except for extremely short lifetimes.

Figure 10 illustrates the contribution of different spin relaxation channels to the total relaxation. A summary of decoherence times for different fields and relaxation mechanisms can be found in Section S7 of the Supporting Information. The results show again that the dominant relaxation pathway is due to the g-anisotropy relaxation.^81^ The effect of the g-anisotropy mechanism on the triplet yield scales with the square of the applied magnetic field intensity (see eq 17) and therefore markedly impacts the spin dynamics at large applied magnetic fields; the hyperfine relaxation mechanism and the spin rotational mechanism turn out to have a rather negligible impact on spin dynamics in the studied system. The weak influence of the hyperfine and spin rotational relaxation mechanism on the lipid peroxide radicals in a bilayer stands in contrast to the influence of those spin relaxation channels on free peroxide lipid radicals, where the high mobility of radicals leads to efficient spin relaxation due to the modulation of the hyperfine interactions.

**Figure 10:**
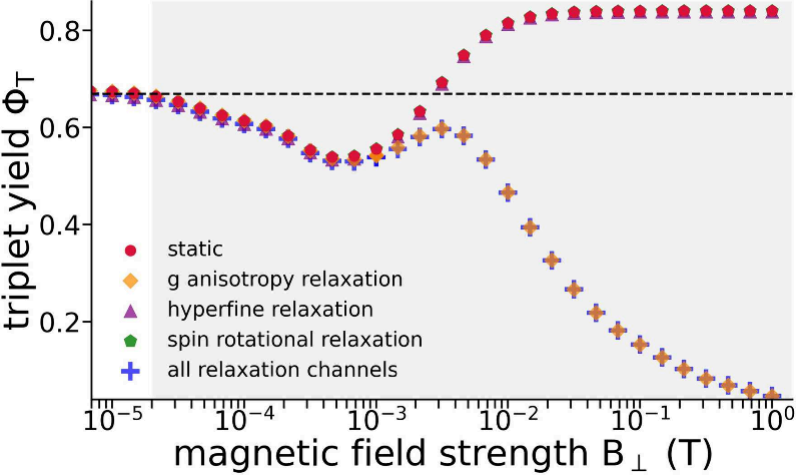
MFE predicted for a RP of lipid peroxide radicals, where the hyperfine interactions of the H313X nucleus were considered and an RP lifetime of 1 μs was assumed. The calculations were carried out without spin relaxation (static) and including selected spin relaxation channels. The orange markers corresponding to the g-anisotropy relaxation signify that only the g-anisotropy spin relaxation channel has been taken into account in that specific calculation; the green markers show the triplet yield calculated with relaxation effects only due to the spin rotational relaxation; the purple markers show the results for which relaxation was considered to only arise from the hyperfine interactions. The blue markers show the triplet yield calculated with all three relaxation channels taken into account.

## Discussion

Although MFEs on lipids have been theoretically predicted and presumably observed in complex cellular systems,^16,45,82^ it remains a question of whether MFEs due to the radical pair mechanism are feasible in lipid membranes. Skepticism was raised on the basis of the assumption that the peroxide lipid radicals would not have hyperfine couplings of sufficient strength to show hyperfine diverted MFEs or that spin relaxation would be too fast as peroxide lipid radicals are involved. In this study, we have once again demonstrated that the first concern is unsubstantiated,^16^ and for the first time show that relaxation due to the modulation of intraradical interactions is slow. In fact, we recognize peroxide lipid radicals in lipid membranes as a system that is, in fact, well poised for MFEs. As the spin density is strongly centered on the peroxide group, only one hyperfine interaction is significant, which turns out to be mostly isotropic and gives rise to a simple spin system *a priori* well-known to deliver large low-field effects.^79,80^ Furthermore, the radicals are sufficiently immobilized to prevent major spin rotational relaxation, and the hyperfine anisotropy is likewise small, suggesting that relaxation processes are slow and the system thus expected to exhibit magnetic field-dependent spin dynamics. Finally, we have observed that the relaxation due to the g-anisotropy mechanism can induce large MFEs at higher field strengths.

While we have not investigated interradical interactions here and expect that the effect will be attenuated as a result of these couplings in the low field, we note that previous studies have suggested that these too could be overcome, for example, by three-radical interactions.^16^ Given our predictions, we strongly encourage MFE studies of lipid membranes of unsaturated phospholipids in simple chemical systems, which are currently still lacking in scope and quality.

The theoretical prediction that MFEs modulate RP chemistry in lipid bilayers could be tested experimentally through a combination of spin-sensitive and structural readouts. Changes in triplet yield, which determine the steady-state abundance of lipid peroxide radicals, can for example be quantified using fluorescence spectroscopy, spin-trapping, and electron paramagnetic resonance (EPR).^83,84^ EPR typically requires ensembles of ≳ 10^7^ spins, but the size of the model lipid bilayer could be scaled up in the experiment. Complementary single-spin sensitivity can be achieved by embedding nitrogen-vacancy (NV) centers in nanodiamonds into model bilayers, enabling direct detection of the spin state and relaxation dynamics of individual lipid peroxide radicals with nanoscale resolution.^85^ In parallel, field-dependent changes in bilayer structure could be assessed, since radical-induced peroxidation alters the packing of the lipid tails and thereby modifies bilayer thickness, area per lipid, and bilayer permeability.^86^ These structural parameters can be probed by small-angle X-ray or neutron scattering,^87^ atomic force microscopy,^88^ and permeability assays.^89^ The concentration of lipid peroxide radicals could be measured through lipidomics^83^ or fluorescent peroxidation probes such as C11-BODIPY, which provide *in situ* measurements.^90^ Following the strategy outlined in Ortiz et al.,^91^ one could measure spin–lattice relaxation times (*T*_1_) in bilayers containing both nonperoxidized and peroxidized lipids in the presence of H_2_O_2_, systematically varying the applied magnetic field instead of oxidant concentration. In such a design, decreases in triplet yield would be reflected by lower peroxide radical concentrations and a corresponding increase in bilayer thickness and decrease in permeability, whereas increases in triplet yield would manifest in the opposite direction. This integrated approach directly connects MFEs to measurable physical and structural changes in lipid bilayers, providing a feasible pathway to experimental verification of the theoretical predictions.

Considering the implication of the findings for systems *in vivo*, lipid peroxidation in cancer cells has become a prominent research topic.^3–15^ Lipid peroxidation can lead to ferroptosis, which is a form of regulated cell death.^25^ In the context of cancer, lipid peroxidation plays a dual role:^12,92–95^ While it has the potential to damage normal cells, leading to cancer initiation,^7,10,17,18,52^ it can also be used for cancer therapy by triggering ferroptosis within malignant cells. Interestingly, cancer cells have been shown to have significantly higher ferroptosis sensitivity than normal cells,^25,96,97^ which could be leveraged for therapy. Targeted cancer therapy approaches using lipid peroxide radicals have been proposed to initiate cell death through ferroptosis in predominantly cancer cells.^5,94,98–100^ Furthermore, the use of magnetic fields to increase ferroptosis through increased lipid peroxidation in cancer cells has been suggested.^13,14,43^

In the present study, we offer a theoretical explanation for MFEs in peroxide lipids that could be used to trigger ferroptosis. The RP mechanism could be used to regulate the amount of lipid peroxide radicals through an external magnetic field, as detailed in Figure 1. An increase in triplet yield would be equivalent to an increase in chain propagation, which could induce ferroptosis. The present investigation shows that if spin relaxation is taken into account, the range of external magnetic field strength which would lead to an increased triplet yield compared to zero field is narrow (see Figure 9B–D). Without spin relaxation effects, one would have expected an increased triplet yield for most RP lifetimes for high magnetic fields, as shown in Figure 9. The results therefore indicate that ferroptosis could potentially increase via the RP mechanism but would require the external magnetic field to be within a rather narrow interval, matched to the coherent lifetime (e.g., ≈10 mT for a lifetime of 100 ns). An alternative would be to expose cancerous tissues to strong magnetic fields over longer periods of time in an attempt to down-regulate the oxidative defense, to then target the cells via chemotherapeutic induced ROS bursts outside of the magnetic field.^101,102^

The use of MFEs in lipid peroxide radicals for diagnostics might offer a wider applicability than the use of MFEs for treatment in cancer therapy. If the MFEs are used for diagnostics, one could also utilize the MFEs that lead to a triplet yield decrease under magnetic fields. Practically, one could measure the radical concentration in a membrane when no magnetic field is present, subsequently expose samples to high magnetic fields, and observe if a difference in peroxide lipid radical concentration appears. If lipid peroxide radicals are more prominent in cancer cells than in normal cells,^94^ a larger difference in the concentration of peroxide lipids in the membranes of cancer cells would be expected.

## Conclusions and Outlook

Peroxide lipids and normal lipids show different dynamic traits. The relative mobility of the two lipid tails is enhanced in lipid peroxide radicals compared to the nonperoxidized lipids (see Figure 4). Moreover, peroxidation induces the lipid tails to bend away from one another and reorient toward the aqueous interface, which increases the average area per lipid in bilayers enriched with lipid peroxide radicals. As a consequence, such bilayers exhibit reduced thickness and elevated water permeability relative to membranes without lipid peroxide radicals.^86^ Since the properties of lipid bilayers depend on the fraction of lipid peroxide radicals incorporated into their structure, these observations indicate that RPM-driven modulation of lipid peroxide radical yields has direct implications for bilayer structural integrity and barrier function. For example, in strong external magnetic fields, the RPM predominantly reduces the triplet reaction yield, thereby lowering the abundance of lipid peroxide radicals within the membrane (see Figure 9), which changes the membrane properties toward those of a nonradicalized bilayer.

Upon the formation of a spin-correlated radical pair of peroxide lipids, the associated spin dynamics are mostly determined by the single hyperfine interaction of the H313X nucleus in weak magnetic fields and the Zeeman interaction, inducing g-anisotropy relaxation in high magnetic fields. The influence of different spin relaxation channels on the triplet yield of the associated RP reaction was investigated to determine if the MFEs predicted under idealized static conditions persist in the lipid bilayer in significant strength when realistic thermal motion is taken into account. Spin relaxation due to changes in the hyperfine coupling and spin rotational relaxation mechanisms, related to the rotation of the peroxide, was found to be insignificant compared to relaxation induced by the g-anisotropy mechanism in high magnetic fields. The influence of spin rotational relaxation turned out to be weak because the rotation of the peroxide group is sufficiently hindered, with a rotational correlation time on the order of 1 ns. The relaxation channel via the hyperfine interaction likewise contributed little to spin dynamics, because the hyperfine H313X tensor is only weakly asymmetric and therefore only induces weak fluctuations of the coupling when modulated by thermal motion. The g-anisotropy relaxation mechanism, in contrast, did have a marked effect on the triplet yield of the RP reaction for high external magnetic fields in RPs. Instead of diminishing MFEs, the g-anisotropy relaxation mechanism turned out to greatly enhance MFEs. Whereby, MFEs are defined as a change in triplet quantum yield and therefore lipid peroxide radical concentration in the bilayer compared to a model lipid bilayer with no external magnetic field applied.

Lipid bilayers with lipid peroxide radicals in human cells are associated with many diseases. Our study suggests that it is not impossible, in principle, that lipid peroxide radical recombination could be modulated through applied magnetic fields, a possibility for future applications in diagnostics and therapy.

In a realistic membrane, more relaxation mechanisms, which have been neglected here, could play a role. An important example is the dipolar interaction between individual peroxide lipid radicals. We have looked at intraradical relaxation in this study because the interradical interactions would require a detailed study of individual lipid encounters, which requires a separate investigation, as encounters of peroxide lipid radicals in the present study were too infrequent to deduce statistically significant information. In future investigations, it will be interesting to take into account spin relaxation effects that arise between pairs or triplets of radicalized lipids.^103^

## Supplementary Material



## Notes

Chemical structure diagrams of the lipids with and without a peroxide group, a figure depicting the workflow to parametrize the lipid peroxide radicals and embed them in a lipid bilayer, the diffusion constants of the peroxide lipids and the diffusion lengths for different radical pair lifetimes, an illustration of the height of the peroxide groups in the membrane patch, plots of the minimal distance between two peroxide lipids over the simulation time, the correlation times for the dihedral angle rotation, the coherence times for different relaxation mechanisms, the movement of the bond between the lipid tail and the peroxide group, dipolar coupling, hyperfine coupling and the change in g-tensors, a comparison of the isotopic hyperfine coupling distributions obtained from structures from the MD simulation and obtained though the mapping procedure of the dihedral angle, the average hyperfine tensor of the H13X nucleus is given as well as the average g-tensor of the 12 peroxide lipids, the covariance matrices of the average hyperfine and g-tensors are given and a plot showing the similarity in triplet quantum yield for an external magnetic field perpendicular and parallel to the membrane patch (PDF)
