# Supplementary material for: Spin Relaxation Does Not Preclude Magnetic Field Effects on Lipid Autoxidation
Source: ACS Cent Sci. 2025 Dec 21;12(1):49–62. doi: 10.1021/acscentsci.5c01229 (PMC12856652; doi:10.1021/acscentsci.5c01229)
Supplement: Supplementary file 1 [file oc5c01229_si_001.pdf]

Spin relaxation does not preclude magnetic field effects on lipid autoxidation (Supplementary Information)

Gesa Grüning,[*,†,‡,¶] Luca Gerhards,[†] Chris Sampson,[§,∥] Daniel R. Kattnig,[§,∥] and Ilia A. Solov'yov[*,†,⊥,#]

†*Institute of Physics, Carl von Ossietzky University, Carl-von-Ossietzky-Str. 9-11, 26129 Oldenburg, Germany*

‡*School of Physics, University of New South Wales, Sydney, NSW, 2052, Australia*

¶*School of Biotechnology and Biomolecular Sciences, University of New South Wales, Sydney, NSW, 2052, Australia*

§*Living Systems Institute, University of Exeter, Stocker Road, EX4 4QD, United Kingdom*

∥*Department of Physics, University of Exeter, Stocker Road, EX4 4QL, United Kingdom*

⊥*Research Center for Neurosensory Science, Carl von Ossietzky Universität Oldenburg, 26111 Oldenburg, Germany*

#*Center for Nanoscale Dynamics (CENAD), Carl von Ossietzky Universität Oldenburg, Institut für Physik, Ammerländer Heerstr. 114-118, 26129 Oldenburg, Germany*

E-mail: g.gruning@unsw.edu.au; ilia.solovyov@uni-oldenburg.de

Contents

1 Parametrization of the lipid peroxide radicals **S3**

2 Visualization of the peroxide groups in the lipid bilayer **S5**

3 Lateral diffusion of the lipid peroxide radicals **S6**

4 Radical distances **S7**

5 Correlation times **S9**

5.1 Dihedral angle Ω rotation S9

5.2 Motion of the bond between the lipid tail and the peroxide group S9

5.3 Hyperfine coupling tensor correlation times S10

5.4 g-tensor correlation times S10

5.5 Dipolar tensor auto-correlation times S13

6 Coherence times **S13**

7 Isotropic hyperfine coupling component from MD and dihedral angle matching method **S16**

8 Average hyperfine coupling tensor **S17**

9 Average g-tensor **S17**

10 Covariance matrix of the hyperfine coupling tensor **S18**

11 Covariance matrix of the g-tensor **S19**

12 Triplet reaction yield for different magnetic field orientations and calculation methods **S20**

1 Parametrization of the lipid peroxide radicals

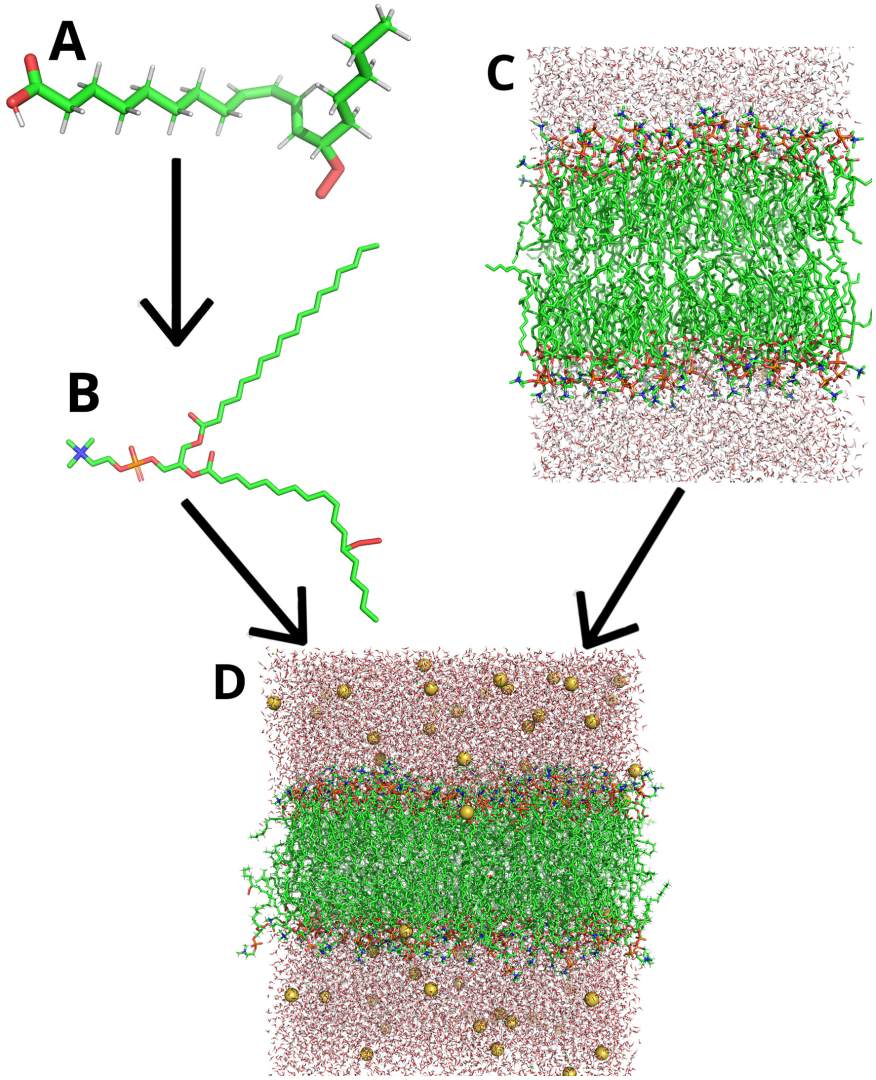

Figure S1: Parametrization workflow: The lipid tail with the peroxide group was created with the package Molefacture in VMD[1] (**A**) and the structure was optimized using the VMD package Force Field Toolkit (ffTK)[2] which in turn relies on gaussian09[3,4] and NAMD.[5,6] A lipid peroxide radical was created in Molefacture by replacing one of the two identical tails of 1,2-Dioleoyl-sn-Glycero-3-Phosphocholine with the peroxidized tail (**B**). A homogeneous membrane patch with 255 1-Palmitoyl-2-linoleoyl-sn-glycero-3-phosphocholine lipids surrounded by water molecules was created in VMD (**C**). 12 of the PLPC lipids were substituted with the lipid peroxide radicals created in **B**. Furthermore, the membrane patch was neutralized with Na^+ and Cl^- in VMD and equilibrated using NAMD[5,6] (**D**).

The lipid peroxide radical was parametrized using the VMD[1] packages Molefacture[7] and the Force Field Toolkit (ffTK).[2] First, the structure of one lipid tail with a peroxide group attached at the C313 atom was created in Molefacture (see Fig. S1**A**). Next, a topology file was created using Molefacture and force field parameters were calculated using the ffTK. The ffTK used the structure optimization implemented in Gaussian09[3] to find the correct bonded and non-bonded interactions between the atoms, the atomistic charges and the correct bond lengths, angles and dihedrals in the lipid tail with the attached peroxide group. Subsequently, the optimized structure of the peroxidized lipid tail substituted the non-peroxidized lipid tail in 1,2-Dioleoyl-sn-Glycero-3-Phosphocholine (see Fig. S1**B**) in Molefacture. Next, a lipid membrane with 255 non-peroxidized lipids (1-Palmitoyl-2-linoleoyl-sn-glycero-3-phosphocholine) was created in VMD (see Fig. S1**C**). Finally, 12 of the PLPC lipids in the membrane patch were substituted with the lipid peroxide radicals created in Fig. S1**B**. The membrane patch was then neutralized with Na^+ and Cl^-, where the resulting topology files were generated using the VMD package psfgen (see Fig. S1**D**).

2 Visualization of the peroxide groups in the lipid bilayer

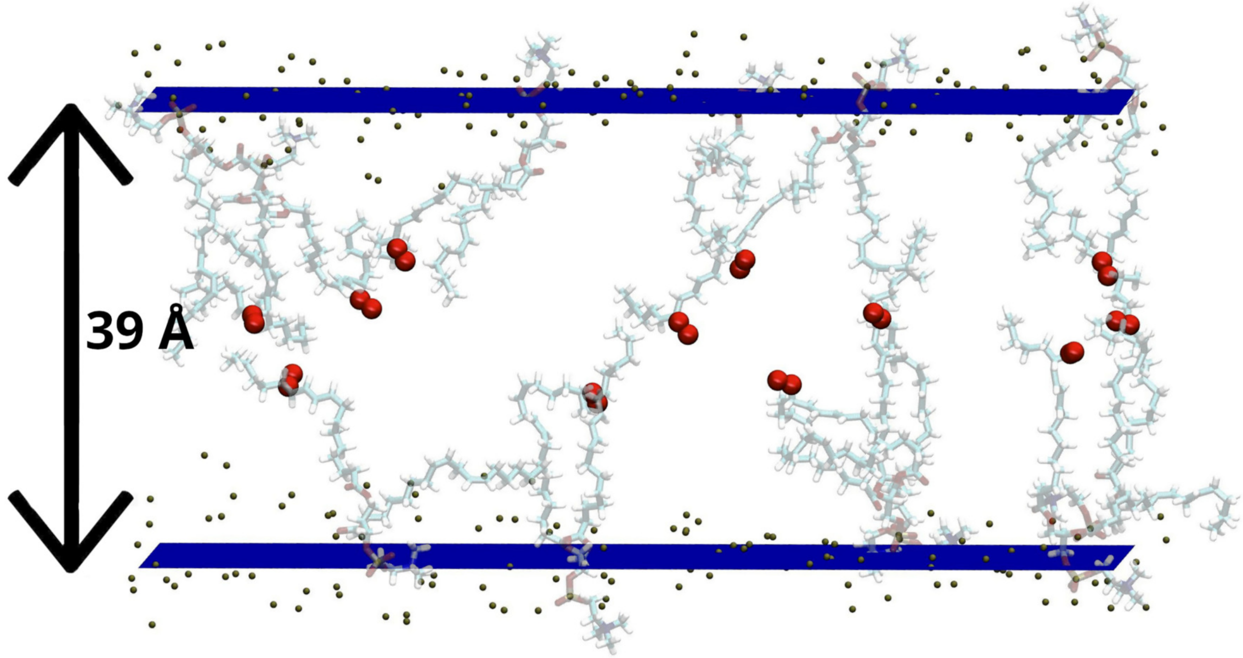

Figure S2: The schematic depth of the peroxide group (red spheres) in the studied membrane patch. The outer layers of the membrane are defined by planes passing through the phosphate atoms (ochre spheres) in the lipid head groups. The lipid tails of the lipid peroxide radicals are shown as transparent.

3 Lateral diffusion of the lipid peroxide radicals

The lateral diffusion constant D of each lipid can be calculated via the free Brownian diffusion length L_D:[8,9]

$$D = \frac{L_D^2}{4t_{sim}} \tag{S1}$$

where L_D measures the mean displacement of each lipid peroxide radical represented through the displacement of the phosphate atom in the head group over the time of the simulation t_{sim}, which is 1,360 ns in the present study.

Table S1 summarizes the lateral diffusion constants for the 12 lipid peroxide radicals. The average diffusion constant for the 12 lipid peroxide radicals is $\langle D \rangle = 2.7\ \mu\text{m}^2/\text{s}$. The individual calculated diffusion constants between 0.7 - 8.5 $\mu\text{m}^2/\text{s}$ agree with previous publications that estimated the values[10] or measured D experimentally[9] in the range of 1.4 - 7 $\mu\text{m}^2/\text{s}$ in lipids in giant uni-lamellar vesicles.

The average diffusion constant $\langle D \rangle = 2.7\ \mu\text{m}^2/\text{s}$ indicates that a lipid peroxide radical moves on average 10.4 Å during a radical pair lifetime of 100 ns, 32.9 Å during a RP lifetime of 1 μs and 103.9 Å during a RP lifetime of 10 μs.

Table S1: Diffusion lengths L_D and lateral diffusion constants D of the lipid peroxide radicals. L_D was measured from the displacement of the lipid head group in the MD simulation and D was calculated using L_D in Eq. (S1).

radical	L_D (Å)	D ($\frac{\mu\text{m}^2}{\text{s}}$)	radical	L_D (Å)	D ($\frac{\mu\text{m}^2}{\text{s}}$)
lipid 1	28.1	1.5	lipid 7	43.4	3.5
lipid 2	51.2	4.8	lipid 8	19.1	0.7
lipid 3	33.5	2.1	lipid 9	21.1	0.8
lipid 4	36.7	2.5	lipid 10	41.3	3.1
lipid 5	29.8	1.6	lipid 11	24.6	1.1
lipid 6	67.9	8.5	lipid 12	37.4	2.6

4 Radical distances

In total, the lipid bilayer contains 12 lipid peroxide radicals. Figure S3 shows the distance of each lipid peroxide radical to the nearest of the 11 other lipid peroxide radicals over time. To enable the F-pair mechanism, two radicals have to come into a reasonably close contact distance. Figure S3 shows that this is indeed the case for the lipid peroxide radicals considered in the present MD simulation. Figure S4 shows that the average minimal distance between two lipid peroxide radicals is on the order of below 20Å.

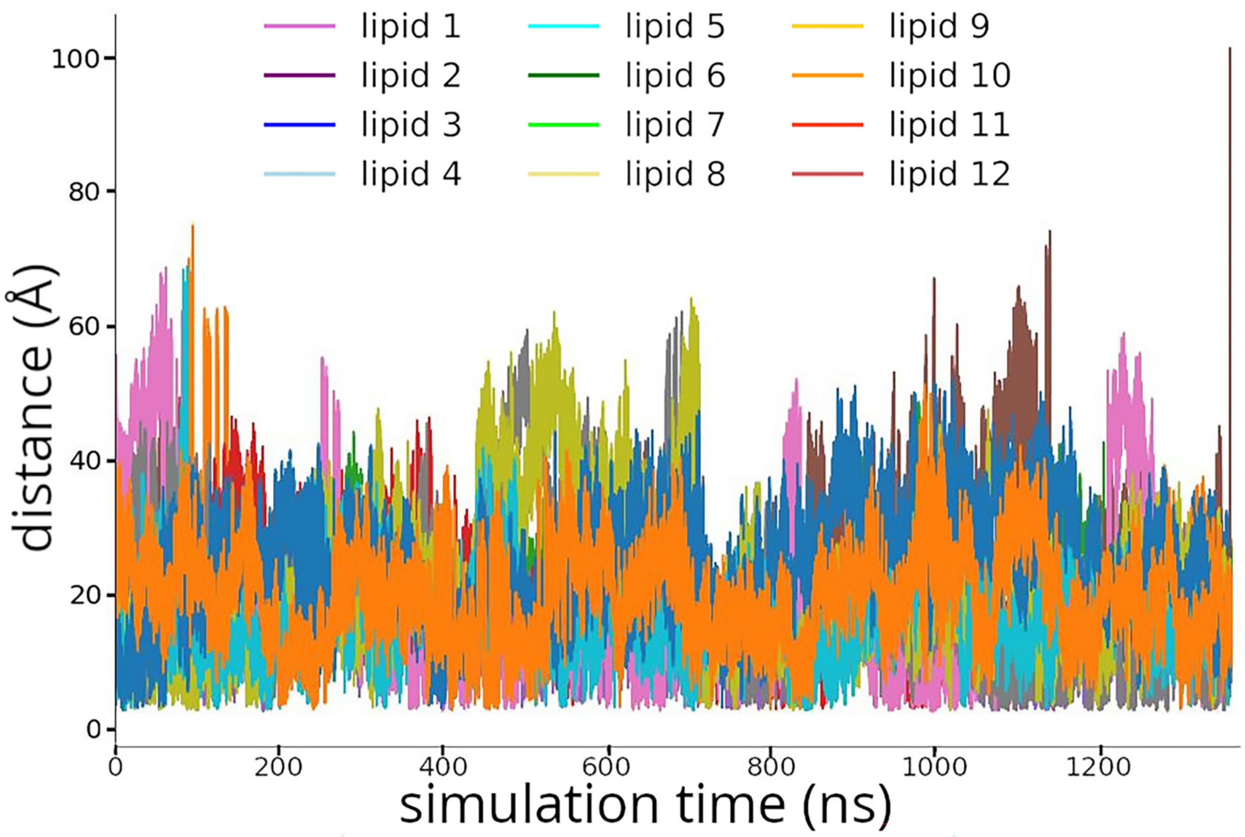

Figure S3: The minimal distance between a lipid peroxide radical and its nearest radical neighbor. The distances are measured at the O_{13A} nucleus of the peroxide group.

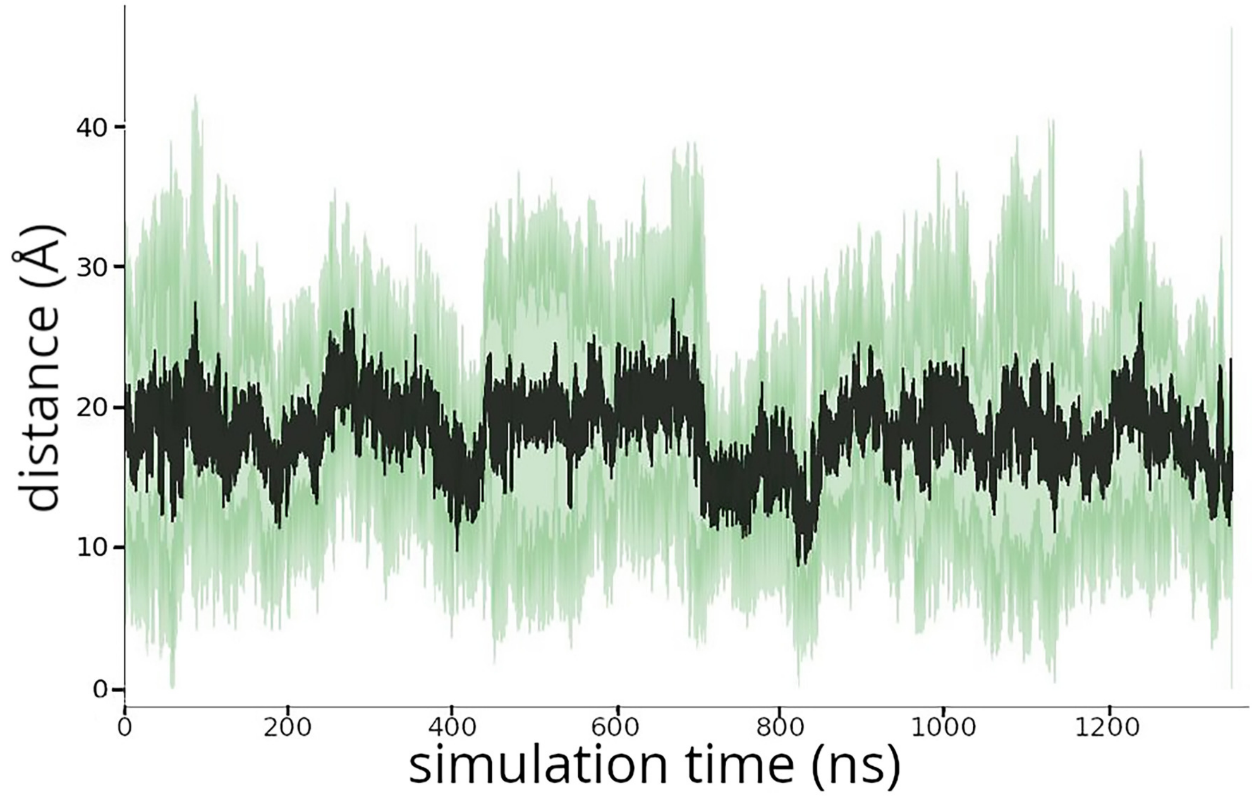

Figure S4: The average minimal distance between two lipid peroxide radicals (black) with standard deviation (light green).

5 Correlation times

5.1 Dihedral angle Ω rotation

Table S2: Effective correlation times of the rotation of the peroxide group – quantified by the change in the dihedral angle Ω – for the 12 lipid peroxide radicals in ns.

radical	τ^{Ω} (ns)	radical	τ^{Ω} (ns)
lipid 1	0.3	lipid 7	0.2
lipid 2	1.0	lipid 8	0.7
lipid 3	0.3	lipid 9	0.3
lipid 4	1.0	lipid 10	0.9
lipid 5	3.4	lipid 11	0.8
lipid 6	2.2	lipid 12	0.7

The average correlation time of the rotation of the peroxide group quantified by the change of the dihedral angle is $\bar{\tau}^{\Omega} = 0.97$ ns.

5.2 Motion of the bond between the lipid tail and the peroxide group

Table S3: Effective correlation times τ^{γ} of the movement of the peroxide group were quantified by the movement of the vector $\overline{\mathrm{O13AC313}}$ from the O13A nucleus to the C313 nucleus. C313 is the carbon atom on the lipid tail where the peroxide group is attached through the bond to O13A. The movement of the bond is measured through the cosine of the angle γ between the vector $\overline{\mathrm{O13AC313}}(t_0)$ at the first time instance t_0 of the MD simulation and the vector $\overline{\mathrm{O13AC313}}(t_i)$ at a time instance t_i.

radical	τ^{γ} (ns)	radical	τ^{γ} (ns)
lipid 1	2.1	lipid 7	2.2
lipid 2	2.6	lipid 8	3.3
lipid 3	4.2	lipid 9	6.8
lipid 4	2.8	lipid 10	8.2
lipid 5	2.2	lipid 11	1.4
lipid 6	4.7	lipid 12	2.9

The average correlation time of the movement of the peroxide bond quantified by the angular displacement of the OC bond is $\bar{\tau}^{\gamma} = 3.6$ ns.

5.3 Hyperfine coupling tensor correlation times

Table S4 shows the effective correlation times of the hyperfine coupling tensor of the H313X nucleus in the 12 lipid peroxide radicals and the average correlation time for each auto- and cross-correlation time of the hyperfine tensor components. As expected, the average auto-correlation times are longer than the average cross-correlation times (see last column Table S4). This condition serves as a sanity check: if the cross-correlation times are longer than the auto-correlation times, the approximations necessary for Redfield theory might break down.[11]

5.4 g-tensor correlation times

Table S5 shows the effective correlation times of the g tensor anisotropy in the 12 lipid peroxide radicals and the average correlation time for each auto- and cross-correlation time of the g tensor components.

Table S4: Effective correlation times τ^h in ns computed for the matrix elements of the hyperfine coupling tensor of the H313X nucleus in the 12 lipid peroxide radicals and the average correlation time $\bar{\tau}^h$. The τ^h values were obtained by fitting Eq. (6) to the correlation function of the respective hyperfine tensor matrix element.

H. tensor comp.	τ^h(L1) ns	τ^h(L2) ns	τ^h(L3) ns	τ^h(L4) ns	τ^h(L5) ns	τ^h(L6) ns	τ^h(L7) ns	τ^h(L8) ns	τ^h(L9) ns	τ^h(L10) ns	τ^h(L11) ns	τ^h(L12) ns	$\bar{\tau}^h$ ns
A_{xx}, A_{xx}	0.9	0.7	0.7	2.2	0.4	0.7	1.8	0.6	0.7	1.3	0.4	0.6	0.9
A_{xx}, A_{xy}	0.0	0.2	0.1	1.4	0.8	0.8	1.0	0.0	0.0	0.1	0.1	0.0	0.4
A_{xx}, A_{xz}	1.4	0.3	0.0	0.0	0.2	0.0	0.0	0.0	0.4	0.0	0.0	1.2	0.3
A_{xx}, A_{yy}	0.1	1.2	0.2	0.2	0.2	0.2	0.1	0.2	0.1	0.7	0.1	0.2	0.3
A_{xx}, A_{yz}	0.9	0.7	0.2	0.0	0.0	1.1	0.0	0.1	0.0	0.0	1.1	0.7	0.4
A_{xx}, A_{zz}	0.5	0.3	0.5	2.1	0.2	0.6	0.3	0.4	0.5	0.4	0.2	0.3	0.5
A_{xy}, A_{xy}	1.4	2.6	1.2	0.4	0.7	2.1	0.7	0.6	0.6	1.1	1.6	0.5	1.1
A_{xy}, A_{xz}	0.5	0.5	0.0	0.1	0.4	0.0	0.1	0.1	0.1	0.0	0.1	0.9	0.2
A_{xy}, A_{yy}	0.1	0.4	0.0	0.2	0.7	0.2	0.2	0.1	0.0	0.0	0.1	0.0	0.2
A_{xy}, A_{yz}	1.0	0.0	0.2	0.9	0.0	0.0	0.1	0.0	0.3	0.0	0.0	0.2	0.2
A_{xy}, A_{zz}	0.2	0.4	0.8	0.0	1.1	0.0	0.2	0.1	0.0	0.1	0.1	0.0	0.3
A_{xz}, A_{xz}	1.4	0.8	2.9	0.6	0.5	1.4	0.7	0.7	0.6	0.7	0.8	0.6	1.0
A_{xz}, A_{yy}	0.0	0.0	0.0	0.0	0.0	2.0	0.0	0.3	0.0	0.0	0.0	0.0	0.2
A_{xz}, A_{yz}	0.2	0.0	0.0	0.2	0.0	0.1	0.2	0.2	0.2	0.8	0.2	0.8	0.2
A_{xz}, A_{zz}	1.5	0.0	0.0	0.2	0.1	0.0	0.0	0.2	0.0	0.0	0.0	0.2	0.2
A_{yy}, A_{yy}	0.7	1.0	0.8	0.7	0.7	0.5	0.7	0.9	1.0	2.5	2.3	0.7	1.0
A_{yy}, A_{yz}	0.0	0.2	0.0	0.1	0.0	0.0	0.0	0.0	0.4	0.0	0.0	0.0	0.1
A_{yy}, A_{zz}	0.5	0.8	1.4	1.1	0.6	0.3	0.4	0.5	1.0	0.5	0.4	0.9	0.7
A_{yz}, A_{yz}	1.6	0.7	0.6	1.1	0.6	0.7	0.7	1.2	0.7	2.6	0.5	1.2	1.0
A_{yz}, A_{zz}	0.2	0.1	0.8	0.0	0.0	0.0	0.1	0.1	0.0	0.9	0.1	0.1	0.2
A_{zz}, A_{zz}	0.8	1.1	0.7	1.6	0.6	3.3	2.1	0.8	0.8	1.0	0.8	0.7	1.2

Table S5: Effective correlation times τ^{g} in ns computed for the matrix elements of the g-tensor of the 12 lipid peroxide radicals and the average correlation time $\bar{\tau}^{\mathrm{g}}$ of the respective matrix elements. The τ^{g} values were obtained by fitting Eq. (12) to the correlation function of the respective g-tensor matrix element.

g-tensor components	τ^{g}(L1) ns	τ^{g}(L2) ns	τ^{g}(L3) ns	τ^{g}(L4) ns	τ^{g}(L5) ns	τ^{g}(L6) ns	τ^{g}(L7) ns	τ^{g}(L8) ns	τ^{g}(L9) ns	τ^{g}(L10) ns	τ^{g}(L11) ns	τ^{g}(L12) ns	$\bar{\tau}^{\mathrm{g}}$ ns
A_{xx}, A_{xx}	2.1	1.0	0.8	1.4	0.5	2.9	0.9	0.6	1.2	0.9	1.4	0.8	1.2
A_{xx}, A_{xy}	0.0	0.2	0.0	0.0	0.0	2.5	0.7	0.1	0.1	0.5	0.1	0.0	0.4
A_{xx}, A_{xz}	0.0	0.0	0.0	1.5	0.1	0.0	0.1	0.1	0.2	0.0	0.0	0.8	0.2
A_{xx}, A_{yy}	1.0	0.7	0.3	0.7	1.9	0.6	1.0	0.2	2.9	0.5	0.3	0.5	0.9
A_{xx}, A_{yz}	0.2	0.0	0.0	0.0	1.1	0.4	1.0	0.1	0.0	0.3	0.0	0.4	0.3
A_{xx}, A_{zz}	0.3	0.3	0.6	0.3	0.3	0.3	1.2	1.2	0.4	0.5	2.5	0.3	0.7
A_{xy}, A_{xy}	1.3	2.8	0.8	1.5	0.9	4.9	2.0	0.7	0.8	5.0	0.8	0.8	1.9
A_{xy}, A_{xz}	0.2	0.0	0.0	0.0	0.0	0.8	0.0	0.2	0.1	0.0	0.0	0.0	0.1
A_{xy}, A_{yy}	0.0	3.3	0.0	0.7	0.7	0.4	0.2	0.1	0.0	0.0	1.6	1.3	0.7
A_{xy}, A_{yz}	0.0	0.0	0.4	0.7	0.2	0.1	0.3	0.0	0.0	0.1	0.2	0.0	0.2
A_{xy}, A_{zz}	0.0	0.0	0.0	1.1	0.6	0.0	1.3	0.0	0.1	0.3	0.2	0.0	0.3
A_{xz}, A_{xz}	2.3	0.5	0.9	0.7	1.8	1.5	0.6	0.6	0.7	0.8	0.9	0.6	1.0
A_{xz}, A_{yy}	0.0	2.2	0.1	1.0	2.2	0.0	0.1	0.0	1.3	0.0	0.1	0.0	0.6
A_{xz}, A_{yz}	0.0	1.1	0.4	0.0	0.1	0.0	0.0	0.0	0.1	0.0	0.0	0.0	0.2
A_{xz}, A_{zz}	0.3	0.0	0.0	0.3	0.1	0.0	0.1	0.1	0.0	0.0	0.0	0.0	0.1
A_{yy}, A_{yy}	1.6	1.2	0.6	0.9	1.3	1.8	0.8	1.1	0.5	1.3	0.8	0.8	1.1
A_{yy}, A_{yz}	0.1	0.0	0.0	0.1	0.0	0.0	2.0	0.1	0.0	0.6	0.0	0.3	0.3
A_{yy}, A_{zz}	0.7	0.4	0.2	3.4	0.5	2.6	0.4	0.7	0.3	0.4	0.3	0.5	0.9
A_{yz}, A_{yz}	2.5	0.7	0.6	2.5	1.3	2.3	3.0	0.9	0.8	0.9	0.5	0.5	1.4
A_{yz}, A_{zz}	0.0	0.0	0.2	0.0	0.0	0.0	0.0	0.1	0.1	0.1	0.2	0.0	0.1
A_{zz}, A_{zz}	0.7	0.6	0.7	1.0	0.8	1.4	2.0	0.8	0.5	0.8	1.6	0.6	1.0

5.5 Dipolar tensor auto-correlation times

Table S6: Effective auto-correlation times of the dipolar coupling in the 12 lipid peroxide radicals in ns.

Dipolar tensor component	L1 ns	L2 ns	L3 ns	L4 ns	L5 ns	L6 ns	L7 ns	L8 ns	L9 ns	L10 ns	L11 ns	L12 ns
A_{xx}	1.6	0.8	1.6	1.7	0.9	1.8	1.3	3.2	1.1	3.5	0.8	1.2
A_{yy}	0.4	0.5	2.8	1.9	4.6	1.3	3.6	1.3	4.0	1.9	0.7	4.0
A_{zz}	1.2	3.8	0.4	4.4	5.3	1.9	0.5	2.7	3.8	3.0	0.6	5.1
A_{xy}	0.6	1.2	1.9	0.6	0.5	0.8	2.3	0.5	1.0	1.2	0.9	0.9
A_{xz}	1.7	1.3	4.2	2.1	1.1	1.8	0.4	2.6	1.1	2.2	2.3	0.9
A_{yz}	0.5	1.4	1.1	0.5	3.0	3.0	1.9	0.9	2.5	2.3	2.4	2.4

6 Coherence times

To approximate the decoherence time through each relaxation mechanism the Redfield generator R is diagonalized, where for each eigenmode λ_i of the generator, a characteristic decay time is defined by $1/\mathrm{Re}(\lambda_i)$. An effective decoherence time is approximated by the median of these decay times, which are shown in Tab. S7. Note that the precise knowledge of the decoherence time of the spin system is not trivial when using relaxation theories such as Bloch–Wangsness–Redfield and Nakajima–Zwanzig theory, since several relaxation channels are formed when constructing the complex matrix structure.

Table S7: Effective decoherence times (inverse median of relaxation rates) of different relaxation mechanisms based on Redfield eigenvalues. In addition, the minimum decay times from the Redfield spectrum are reported.

relaxation mechanism	τ_{eff} (ns)	τ_{min} (ns)
hyperfine	$1.17 \cdot 10^5$	$7.24 \cdot 10^4$
g-anisotropy (10 mT)	$2.78 \cdot 10^2$	$1.74 \cdot 10^2$
g-anisotropy (1 T)	$4.05 \cdot 10^{-2}$	$2.03 \cdot 10^{-2}$

As can be observed, the hyperfine relaxation mechanism has an extraordinarily long

τ_{eff} as the fluctuations of the isotropic interaction with nearby hydrogen nuclei are weak. In contrast, g-anisotropy yields much shorter decoherence times, especially at high fields (> 1 T), with an effective value of 0.041 ns.

An alternative approach to reveal the decoherence time of each relaxation mechanism is a time-dependent evolution of the spin system without a reaction operator that drains density from the spin system. Here, the decoherence time of a selected spin state can be evaluated by fitting an exponential decay function to the time evolution until equilibrium is reached and no oscillations between spin states occur anymore. In Fig. S5, the decoherence time for the g-anisotropy relaxation mechanism using this approach was evaluated for 10 mT.

To further interpret these results we classified each eigenmode according to whether its eigenvector lives mainly on populations (longitudinal relaxation, T_1-like) or on coherences (transverse relaxation, T_2-like). For the g-anisotropy case at 10 mT, the weight of an eigenmode k in the population vs. coherence subspaces is defined as

$$w_{\text{pop}}(k) = \frac{\|P_{\text{pop}} v_k\|^2}{\|v_k\|^2}, \qquad w_{\text{coh}}(k) = 1 - w_{\text{pop}}(k),$$

where v_k is the eigenvector of R and P_{pop} projects onto diagonal density-matrix elements. Modes with $w_{\text{coh}} > 0.9$ are classified as T_2-like, and modes with $w_{\text{pop}} > 0.9$ as T_1-like. This analysis shows that the overwhelming majority of decay channels (over 80%) are T_2-like, with a median $\tau \approx 277$ ns, while the few T_1-like channels are slower ($\tau \approx 1258$ ns). A similar trend can be found for the hyperfine relaxation mechanism. Here, in total 232 modes could be found inheriting a T_2-like character, while only 17 modes are connected to T_1-like behavior. Thus, the effective decoherence times reported in Tab. S7 correspond predominantly to transverse relaxation T_2 of the radical pair, while longitudinal T_1 processes are minor in comparison.

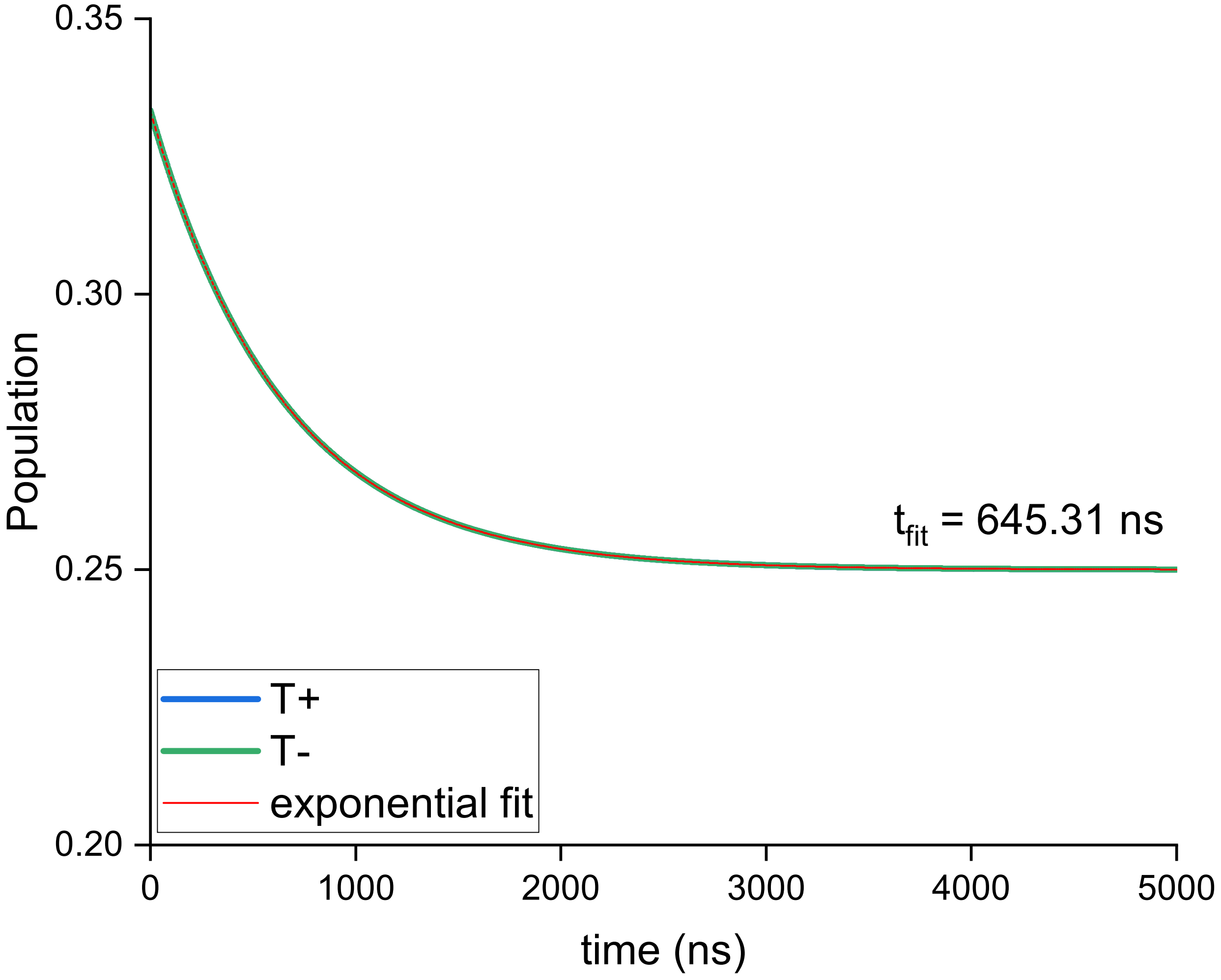

Figure S5: Fit of the decoherence time for the T_- and T_+ population for the g-anisotropy relaxation mechanism at 10 mT. For the fitted decay time, a value of 645.31 ns is found, which is in reasonable agreement with the effective decoherence times in Tab. S7.

7 Isotropic hyperfine coupling component from MD and dihedral angle matching method

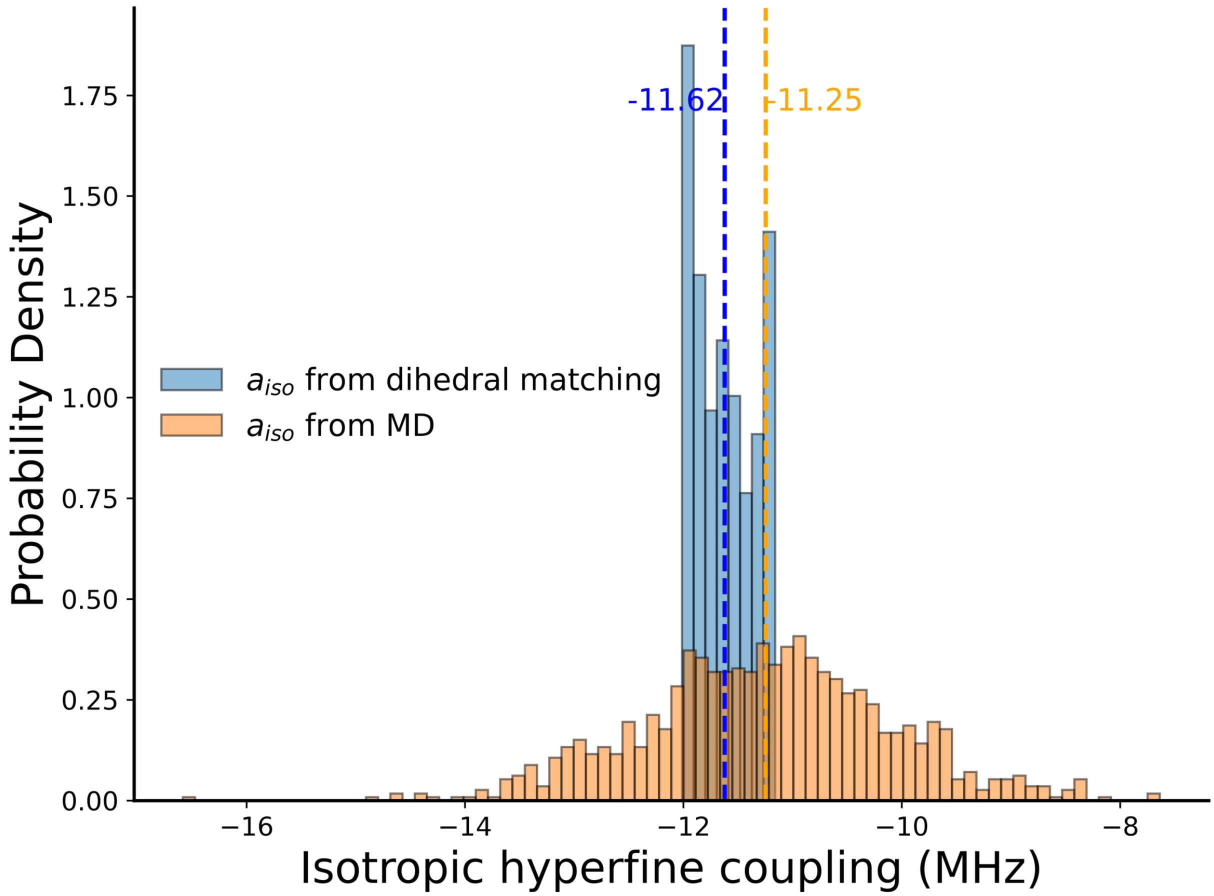

Figure S6: Comparison of the distributions of isotropic hyperperfine coupling component obtaind through DFT calculations (i) based on 1008 structures of the lipid peroxide radicals extracted from the MD simulation (orange), and (ii) based on 16,320,000 structures generated by mapping the hyperfine coupling as a function of the dihedral angle Ω for 12 radicals over 1,360,000 time instances to the position of each lipid peroxide radical in the respective time instance (ligth blue). The distribution (ii) obtained from the dihedral angle matching is a lot narrower, because the matching procedure does not account for structural rearrangements in the lipid tail. The average isotropic hyperfine values, indicated with the dashed vertical lines, are, however, similar for both distributions, and the fluctuations in the conformation of the lipid tail leading to wide spread in (i) are too fast to contribute significantly to spin relaxation effects.

8 Average hyperfine coupling tensor

The hyperfine coupling tensor was extracted from the DFT calculations containing the nuclei selection illustrated in Fig. 2**C** of the main manuscript, which mapped the calculated tensors in the optimized structure to the dihedral angle assumed at each time point. The average hyperfine tensor of the H13X nucleus of the whole MD trajectory and the 12 lipid peroxide radicals in the reference frame of the MD simulation is:

$$\bar{A} = \begin{pmatrix} -11.47 & -1.27\times10^{-02} & -6.58\times10^{-03} \\ -1.27\times10^{-02} & -11.48 & -1.08\times10^{-03} \\ -6.58\times10^{-03} & -1.08\times10^{-03} & -11.78 \end{pmatrix} \text{(mT)}. \tag{S2}$$

9 Average g-tensor

The g-tensor obtained in an analogous method to the hyperfine tensor from the DFT calculations containing the nuclei selection shown in Fig. 2**C** of the main manuscript that mapped the calculated tensors in the optimized structure to the dihedral angle assumed at each time point. The average g-tensor of the whole MD trajectory and the 12 lipid peroxide radicals in the reference frame of the MD simulation was calculated to be:

$$\bar{g} = \begin{pmatrix} 2.0062 & 2.6065\times10^{-03} & -4.3308\times10^{-04} \\ 2.6065\times10^{-03} & 2.0054 & -8.0952\times10^{-07} \\ -4.3308\times10^{-04} & -8.0952\times10^{-07} & 2.0274 \end{pmatrix}. \tag{S3}$$

The diagonal elements of $\bar{g}$ are close to the g value of the free electron $g_e = 2.0023$.

10 Covariance matrix of the hyperfine coupling tensor

The average covariance matrix of the hyperfine tensor $\bar{A}$ with entries in MHz reads as:

$$cov(\bar{A}) =$$

$$\begin{pmatrix} 2.368 & 0.000 & 0.003 & 0.000 & -0.980 & -0.003 & 0.003 & -0.003 & -1.188 \\ 0.000 & 1.721 & -0.008 & 1.721 & 0.017 & -0.001 & -0.008 & -0.001 & -0.016 \\ 0.003 & -0.008 & 1.943 & -0.008 & -0.005 & 0.013 & 1.943 & 0.013 & 0.002 \\ 0.000 & 1.721 & -0.008 & 1.721 & 0.017 & -0.001 & -0.008 & -0.001 & -0.016 \\ -0.980 & 0.017 & -0.005 & 0.017 & 2.410 & 0.003 & -0.005 & 0.003 & -1.219 \\ -0.003 & -0.001 & 0.013 & -0.001 & 0.003 & 1.940 & 0.013 & 1.940 & -0.001 \\ 0.003 & -0.008 & 1.943 & -0.008 & -0.005 & 0.013 & 1.943 & 0.013 & 0.002 \\ -0.003 & -0.001 & 0.013 & -0.001 & 0.003 & 1.940 & 0.013 & 1.940 & -0.001 \\ -1.188 & -0.016 & 0.002 & -0.016 & -1.219 & -0.001 & 0.002 & -0.001 & 2.616 \end{pmatrix} . \quad \text{(S4)}$$

The average covariance matrix was calculated by computing the 12 covariance matrices for the hyperfine coupling in the H13X nucleus in the 12 lipid peroxide radicals and then averaging those covariance matrices.

11 Covariance matrix of the g-tensor

The average covariance matrix of the g-tensor of the 12 lipid peroxide radicals reads as:

$$cov(\bar{g}) =$$

$$\begin{pmatrix} 0.0505 & -0.0003 & 0.0001 & -0.0003 & -0.0209 & -0.0003 & 0.0001 & -0.0003 & -0.0296 \\ -0.0003 & 0.0367 & -0.0003 & 0.0367 & 0.0005 & -0.0002 & -0.0003 & -0.0002 & -0.0001 \\ 0.0001 & -0.0003 & 0.0445 & -0.0003 & -0.0003 & -0.0000 & 0.0445 & -0.0000 & 0.0002 \\ -0.0003 & 0.0367 & -0.0003 & 0.0367 & 0.0005 & -0.0002 & -0.0003 & -0.0002 & -0.0001 \\ -0.0209 & 0.0005 & -0.0003 & 0.0005 & 0.0506 & 0.0002 & -0.0003 & 0.0002 & -0.0296 \\ -0.0003 & -0.0002 & -0.0000 & -0.0002 & 0.0002 & 0.0448 & -0.0000 & 0.0448 & 0.0000 \\ 0.0001 & -0.0003 & 0.0445 & -0.0003 & -0.0003 & -0.0000 & 0.0445 & -0.0000 & 0.0002 \\ -0.0003 & -0.0002 & -0.0000 & -0.0002 & 0.0002 & 0.0448 & -0.0000 & 0.0448 & 0.0000 \\ -0.0296 & -0.0001 & 0.0002 & -0.0002 & -0.0296 & 0.0000 & 0.0002 & 0.0000 & 0.0592 \end{pmatrix}. \tag{S5}$$

12 Triplet reaction yield for different magnetic field orientations and calculation methods

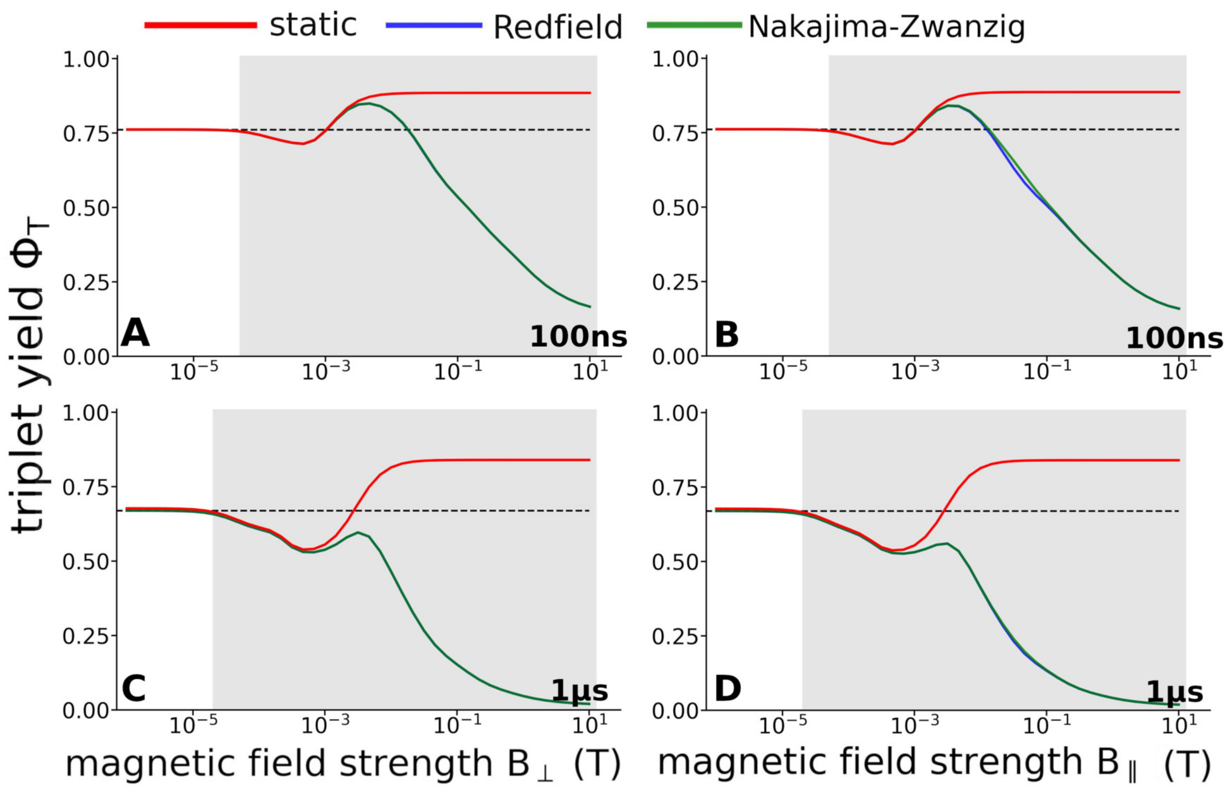

Figure S7: MFE in the lipid bilayer with hyperfine interactions in the H313X nucleus considered without spin relaxation (static) and with spin relaxation taken into account via BRW-theory (Redfield) and using the Nakajima-Zwanzig approach (Nakajima-Zwanzig). **A** and **B** show the results of triplet yield calculations for an RP with a 100 ns lifetime of the RP, while **C** and **D** show the results for an RP with a lifetime of 1 μs. The magnetic field in **A** and **C** is assumed to be directed perpendicular to the membrane surface and in **B** and **D** it is parallel to the surface.

Figure S7 shows that the MFEs and the triplet yield of the studied RP reaction are almost unaffected by the external magnetic field being oriented perpendicular ($B_\perp$) or parallel ($B_\parallel$) to the lipid bilayer surface; the differences between the triplet yields computed for $B_\perp$ and $B_\parallel$ turn out to be observable beyond the second decimal point. The only visual difference

between the resulting triplet yield for $B_\perp$ compared to $B_\parallel$ is that the peak following the trough at about 10 mT field strength is slightly higher for the relaxed case with $B_\perp$ (Fig. S7**A**) than for the relaxed system with $B_\parallel$ (Fig. S7**B**). The physical reason for the insensitivity of the triplet yield to the orientation of the external magnetic field is that the averaged hyperfine interaction of the 12 lipid peroxide radicals turns out to be nearly isotropic (see Fig. 6**C**). Furthermore, Fig. S7 illustrates that the triplet yields calculated using BRW-theory or employing the Nakajima-Zwanzig approach[12–15] are almost identical.

References

(1) Humphrey, W.; Dalke, A.; Schulten, K. VMD - Visual Molecular Dynamics. *J. Mol. Graph. Model.* **1996**, *14*, 33–38.

(2) Mayne, C. G.; Saam, J.; Schulten, K.; Tajkhorshid, E.; Gumbart, J. C. Rapid parameterization of small molecules using the force field toolkit. *J. Comput. Chem.* **2013**, *34*, 2757–2770.

(3) Frisch, M. J. et al. Gaussian˜09 Revision E.01. 2009; Gaussian Inc. Wallingford CT.

(4) Barone, V. *Recent advances in density functional methods*; Chong, D. P.; World Scientific: Singapore, 1995; pp 287–334.

(5) Phillips, J. C.; Braun, R.; Wan, W.; Gumbart, J.; Tajkhorshid, E.; Villa, E.; Chipot, C.; Skeel, R. D.; Kalé, L.; Schulten, K. Scalable molecular dynamics with NAMD. *J. Comput. Chem.* **2005**, *26*, 60–76.

(6) Phillips, J. C. et al. Scalable molecular dynamics on CPU and GPU architectures with NAMD. *J. Chem. Phys.* **2020**, *153*, 044130.

(7) Spivak, M.; Stone, J. E.; Ribeiro, J.; Saam, J.; Freddolino, P. L.; Bernardi, R. C.; Tajkhorshid, E. VMD as a Platform for Interactive Small Molecule Preparation and Visualization in Quantum and Classical Simulations. *J. Chem. Inf. Model.* **2023**, *63*, 4664–4678.

(8) Lindblom, G. In *Encyclopedia of Biophysics*; Roberts, G. C. K., Ed.; Springer Berlin Heidelberg: Berlin, Heidelberg, 2013; pp 1264–1268.

(9) Lipid diffusion in planar membranes investigated by fluorescence correlation spectroscopy. *Biochim. Biophys. Acta, Biomembr.* **2010**, *1798*, 1377–1391.

(10) Sampson, C.; Keens, R. H.; Kattnig, D. R. On the magnetosensitivity of lipid peroxidation: two- versus three-radical dynamics. *Phys. Chem. Chem. Phys.* **2019**, *21*, 13526–13538.

(11) Grüning, G.; Gerhards, L.; Wong, S. Y.; Kattnig, D. R.; Solov'yov, I. A. The Effect of Spin Relaxation on Magnetic Compass Sensitivity in ErCry4a. *ChemPhysChem* **2024**, *25*, e202400129.

(12) Nakajima, S. On Quantum Theory of Transport Phenomena: Steady Diffusion. *Prog. Theor. Phys.* **1958**, *20*, 948–959.

(13) Zwanzig, R. Ensemble Method in the Theory of Irreversibility. *J. Chem. Phys.* **1960**, *33*, 1338–1341.

(14) Fay, T. P.; Lindoy, L. P.; Manolopoulos, D. E. Spin relaxation in radical pairs from the stochastic Schrödinger equation. *J. Chem. Phys.* **2021**, *154*, 084121.

(15) Pažėra, G. J.; Fay, T. P.; Solov'yov, I. A.; Hore, P. J.; Gerhards, L. Spin Dynamics of Radical Pairs Using the Stochastic Schrödinger Equation in MolSpin. *J. Chem. Theory Comput.* **2024**, *20*, 8412–8421.
